# Direct and Inverted Repeats Elicit Genetic Instability by Both Exploiting and Eluding DNA Double-Strand Break Repair Systems in Mycobacteria

**DOI:** 10.1371/journal.pone.0051064

**Published:** 2012-12-10

**Authors:** Ewelina A. Wojcik, Anna Brzostek, Albino Bacolla, Pawel Mackiewicz, Karen M. Vasquez, Malgorzata Korycka-Machala, Adam Jaworski, Jaroslaw Dziadek

**Affiliations:** 1 Institute of Medical Biology, Polish Academy of Sciences, Lodz, Poland; 2 Department of Genetics of Microorganisms, Institute of Microbiology and Immunology, University of Lodz, Lodz, Poland; 3 The University of Texas at Austin, Division of Pharmacology and Toxicology, Dell Pediatric Research Institute, Austin, Texas, United States of America; 4 Department of Genomics, Faculty of Biotechnology, University of Wroclaw, Wroclaw, Poland; Tulane University Health Sciences Center, United States of America

## Abstract

Repetitive DNA sequences with the potential to form alternative DNA conformations, such as slipped structures and cruciforms, can induce genetic instability by promoting replication errors and by serving as a substrate for DNA repair proteins, which may lead to DNA double-strand breaks (DSBs). However, the contribution of each of the DSB repair pathways, homologous recombination (HR), non-homologous end-joining (NHEJ) and single-strand annealing (SSA), to this sort of genetic instability is not fully understood. Herein, we assessed the genome-wide distribution of repetitive DNA sequences in the *Mycobacterium smegmatis*, *Mycobacterium tuberculosis* and *Escherichia coli* genomes, and determined the types and frequencies of genetic instability induced by direct and inverted repeats, both in the presence and in the absence of HR, NHEJ, and SSA. All three genomes are strongly enriched in direct repeats and modestly enriched in inverted repeats. When using chromosomally integrated constructs in *M. smegmatis*, direct repeats induced the perfect deletion of their intervening sequences ∼1,000-fold above background. Absence of HR further enhanced these perfect deletions, whereas absence of NHEJ or SSA had no influence, suggesting compromised replication fidelity. In contrast, inverted repeats induced perfect deletions only in the absence of SSA. Both direct and inverted repeats stimulated excision of the constructs from the *attB* integration sites independently of HR, NHEJ, or SSA. With episomal constructs, direct and inverted repeats triggered DNA instability by activating nucleolytic activity, and absence of the DSB repair pathways (in the order NHEJ>HR>SSA) exacerbated this instability. Thus, direct and inverted repeats may elicit genetic instability in mycobacteria by 1) directly interfering with replication fidelity, 2) stimulating the three main DSB repair pathways, and 3) enticing L5 site-specific recombination.

## Introduction

The first genome of a *Mycobacterium tuberculosis* strain was sequenced in 1998 [1] and since then additional strains, including other species of the *Mycobacterium* genus have been sequenced, with 24 *M. tuberculosis* complete genomes currently available (http://www.ncbi.nlm.nih.gov/genome/genomes/166) [2]–[4]. Sequence analyses of these genomes revealed considerable conservation overall, with as much as 99.95% identity between *M. tuberculosis* and *M. bovis* [4], the main difference being the presence and distribution of repeat elements, including insertion sequences (IS), direct repeats (DR) and mycobacterial interspersed repetitive units, a locus containing variable numbers of tandem repeats (MIRU-VNTR). Indeed, because of the high variability in location and sequence, these and other repetitive elements have served as a tool for molecular typing of *M. tuberculosis* in human isolates [5], [6].

Simple sequence repeats (SSRs), a subset of DNA motifs comprising short tandem repeats of 1–6 nucleotide units, are distributed throughout the mycobacterial genomes at an average of 220–230 tracts per kb [7]. SSRs have been shown to exhibit higher mutation rates than genome-wide average, in both eukaryotes and prokaryotes [8]–[14]. This distinct property has in part been associated with the ability of SSRs to adopt alternative DNA conformations (non-B DNA), in addition to the canonical right-handed B-form [15]–[19], and to promote slipped misalignment and other replication errors during DNA synthesis [20], [21].

Studies over the past few decades have revealed that various types of DNA sequences, in addition of SSRs, may adopt non-B DNA conformations, including DRs (which can form slipped/hairpin structures), inverted repeats (IRs, cruciform structures), polypurine•polypyrimidine tracts with mirror symmetry (triplex conformations), alternating purine-pyrimidine tracts (left-handed Z-DNA) and four closely-spaced runs of 3–5 guanines (G-quadruplexes) [15], [22]–[28]. In addition to a regulatory role during transcription [29]–[32], non-B DNA structures have been implicated in causing genetic instability, including deletions, translocations, and single base substitutions, associated with human disease etiology [16], [22], [33].

The molecular mechanisms underlying the genetic instability induced by repetitive DNA sequences remain to be fully elucidated; however, proteins involved in replication, transcription, and DNA repair pathways have been implicated, by either generating or processing strand breaks in proximity of the repeats [16], [34]–[41]. DNA double-strand breaks (DSBs) lead to cell death if left unrepaired, and homologous recombination (HR) and non-homologous end-joining (NHEJ) are considered the two main pathways for processing DSBs. Thus, the detection of microhomologies at breakpoints of deletions and translocations induced by repetitive DNA has prompted speculation that NHEJ serves as the major pathway for the repair of repetitive DNA-dependent genetic instabilities [30], [42].

Little is known of the genome-wide distribution of repetitive DNA motifs in mycobacteria and their processing by DNA repair systems. We and others have shown that, in addition to HR, mycobacteria possess a prototypical NHEJ apparatus encoded by evolutionarily conserved *ku* and *ligD* genes [41], [43]–[48], as well as a single-strand annealing (SSA) pathway [49]. Herein, we conducted genome-wide *in silico* investigations in *M. smegmatis*, *M. tuberculosis*, and *E. coli* to identify potential non-B DNA-forming repeats, and report that both mycobacterial species are strongly enriched in DRs and IRs. *M. smegmatis* and *E. coli* were used as model systems to investigate the roles of DRs and IRs in inducing deletions, both in wild-type strains and in strains deficient in the common HR pathway or the mycobacterial-specific NHEJ and SSA functions, using both integrated and episomal systems. In *M. smegmatis*, DRs elicited deletions of the intervening sequence even in the absence of functional HR, NHEJ or SSA, whereas both types of repeat induced large deletions, particularly in the DSB repair-deficient strains, in the order ΔNHEJ>ΔHR>ΔSSA. In addition, both DRs and IRs stimulated L5 site-specific recombination at the *attB* site, causing loss of the integrated constructs at much higher frequencies than the non-repeat-containing constructs (which did not carry DR or IR sequences). In *E. coli*, DRs were inert, whereas IRs induced negative supercoiling-dependent and HR-dependent deletions, suggesting a role for non-B DNA structure formation and replication, respectively.

We conclude that, on the one hand, repetitive DNA motifs may pose a challenge to genome stability in mycobacteria, which respond by employing their DSB repair functions. On the other hand, by acting upon a large pool of repetitive sequences, DNA repair may contribute to genomic diversity and adaptation.

## Materials and Methods

### Bacterial strains and cell culture conditions

The bacterial strains are listed in Table S1. Mycobacterial strains were cultured in 8 g/L NB (Nutrient Broth, Difco) and 10 g/L glucose. As required, further additions included: 0.2% Tween-80 (pH 6.0–6.2), 50 µg/mL hygromycin (Hyg), 7.5 µg/mL gentamycin (Gm, 25 µg/mL if used together with kanamycin), and 25 µg/mL kanamycin (Km). *E. coli* strains were cultured in LB media (10 g/L trypton, 5 g/L yeast extract, 10 g/L NaCl, pH 7.0). As required, further additions included 100 µg/mL ampicillin (Amp), 200 µg/mL Hyg, 10 µg/mL Gm (50 µg/mL if used together with Km) and 50 µg/mL Km.

### Cloning

**Figure 1:**
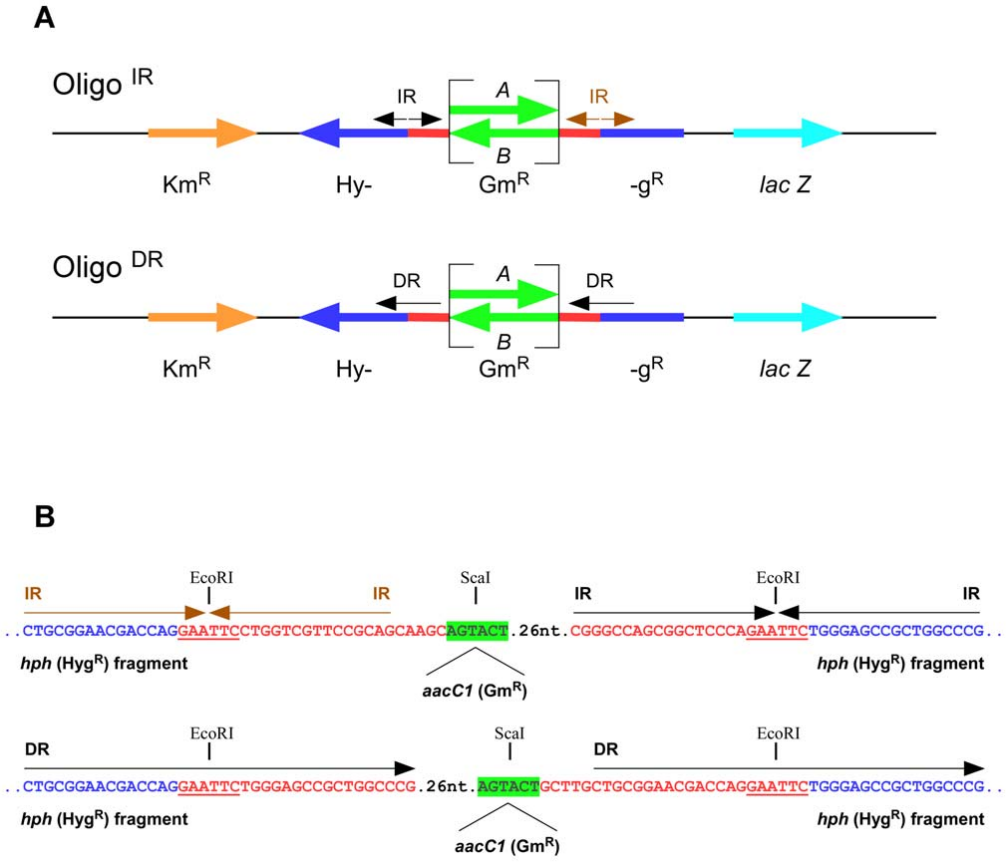
Key features of integration and self-replicating constructs. *Panel A*, Oligo^IR^ and Oligo^DR^ constructs. The *hph* (Hyg^R^, *dark blue*) gene was interrupted by two pairs of IRs (*top*) or one pair of DRs (*bottom; black* and *maroon arrows* over *blue/red*) flanking the *aacC1* (Gm^R^, *green*) gene in both orientations (A and B). These cassettes were further flanked by the *aph* (Km^R^, *orange*) gene on the left and the *lac*Z (blue color, *light blue*) gene on the right. *Panel B*, detailed DNA sequence of the IRs and DRs and the relevant restriction sites (EcoRI and ScaI) used during cloning. Note that because the sequence is given in the 5′→3′ direction with respect to the *hph* gene, the map is opposite to that shown in Panel A.

Constructs were engineered on the basis of the pMV206Km *E. coli/Mycobacterium* shuttle vector and the pMV306Km mycobacterial integration vector (Figure S1). Specifically, the *hph* gene (Hyg^R^) was disrupted by cloning synthetic oligonucleotides, comprising either two pairs of IRs or two DR units, within the EcoRI site of the gene using standard protocols. DRs and IRs were further separated by cloning the *aacC1* gene (Gm^R^) in orientations A and B. This resulted in two pairs of IRs or one pair of DRs, on either side of the *aacC1* gene. The disrupted *hph* was also flanked, 5′ and 3′ respectively, by the *aph* (Km^R^) and *lac*Z (conferring blue color) genes (Figure 1). This yielded two sets of plasmids: pDR^I^A, pDR^I^B, pIR^I^A, pIR^I^B and pDR^E^A, pDR^E^B, pIR^E^A, pIR^E^B (^I^, integrated; ^E^ episomal) (Table S2). The plasmid sequencing data have been deposited in GenBank with the following accession numbers: BankIt1574770 pDR^E^A JX993915, BankIt1574770 pDR^E^B JX993916, BankIt1574770 pDR^I^A JX993917, BankIt1574770 pDR^I^B JX993918, BankIt1574770 pIR^E^A JX993919, BankIt1574770 pIR^E^B JX993920, BankIt1574770 pIR^I^A JX993921, BankIt1574770 pIR^I^B JX993922.

Constructs were used to engineer *E. coli* and mycobacterial strains. The plasmids, or chromosomal DNA, of mycobacterial transformants were recovered and analyzed as described [50]. To determine the extent to which the IR and DR sequences induced genetic instability, PCR analyses were performed to identify loss-of-function of the *aacC1* and *hph* genes. In addition, PCR analyses and Southern blot hybridization were employed to assess the instabilities at the *attB* region of the mycobacterial genome. For the Southern blots, a hybridization probe was generated by PCR and labeled by a non-radioactive primer extension system (DIG-labeling system, Amersham). The primers used for PCR amplification are listed in Table S3.

To perform unmarked deletions of *recBCD* genes in *M. smegmatis* we used a suicidal recombination delivery vector based on p2NIL. The recombination vector carried the region upstream from *recC* together with its 5′ end and the region downstream of *recD* with its 3′ fragment cloned together (Figure S2). Finally, the PacI screening cassette from pGOAL17 was inserted into the constructs, resulting in the suicide delivery vector pMK212, which was then used to engineer the ΔrecBCD *M. smegmatis* strain (Table S1), as described previously [51], [52]. The resultant mutant strain was verified by PCR and Southern blot hybridization (Figure S2).

### Protocol for assessing genetic instabilities caused by DR and IR sequences in *M. smegmatis* and *E. coli*

Constructs (Figures 1 and S1) were introduced into the appropriate *M. smegmatis* and *E. coli* strains and blue, Km^R^ and Gm^R^ colonies were selected. Plasmid instability was determined on non-selective plates by overnight (*E. coli*) or 48 h (*M. smegmatis*) growth of Km^R^, Gm^R^ and blue colony-forming units (CFUs) on LB/NB agar plates, which were then washed-out and plated at the appropriate dilutions on LB/NB agar plates supplemented with either 5-bromo-4-chloro-3-indoxyl-beta-D-galactopyranoside (X-gal) or Hyg. CFUs from X-gal plates were further transferred by replica plating on four LB/NB agar plates that contained the following indicators: 1) Km plus Hyg; 2) Km plus Gm; 3) Km alone and 4) X-gal (Figure S3). The number of ensuing individual CFUs was counted and used for statistical analyses. The frequencies of mutations from 3–6 experiments were analyzed statistically using either the χ^2^ test on 2×2 contingency tables, the z-test or the pair-wise Holm-Sidak test. The Holm-Sidak test was used for the frequency data with standard deviations. In the case where the data were obtained from replica plating, the low numbers of CFUs per plate were cumulatively added (comparing to about 1,000 CFUs from the plates with no selection) and analyzed by the chi square test or the z-test, since these allow comparisons between observed and expected observations.

### Identification of repeat elements in bacterial genomes

Putative non-B DNA-forming motifs, including tandem repeats, DRs, IRs, R•Y tracts and quadruplex-forming motifs were identified in the genomes of *M. tuberculosis*, *M. smegmatis*, and *E. coli* using the criteria reported in Table S4. In the case of IRs, the spacing between repeats was set to ≤20 bases, since cruciform formation tends to be discouraged by large loops (i.e. the bases separating the repeats). By contrast, no minimum loop size is known to prevent direct repeats from interacting; therefore, we allowed any space between DRs. The complete genome sequences of *M. tuberculosis* H37Rv (NC_000962), *M. smegmatis str.* mc^2^155 (NC_008596), and *E. coli str.* K-12 *substr.* MG1655 (NC_000913) were downloaded from the National Center for Biotechnology Information (NCBI) ftp server at ftp://ftp.ncbi.nih.gov/genomes/Bacteria/. Tandem repeats were analyzed by the mreps software [53], DRs were identified according to the repeat-match algorithm of the MUMmer package [54], IRs were screened with the palindrome program of the EMBOSS package [55] and the R•Y and quadruplex-forming motifs were identified using in-house Perl scripts. To determine whether the numbers and lengths of repeats were greater than expected by chance, we compared the *observed* distributions with those *predicted* by reshuffling the respective genome sequences 1,000 times using shuffleseq (EMBOSS).

## Results

### DRs and IRs are overrepresented in mycobacterial genomes

Repetitive DNA sequences, including tandem repeats, DRs, IRs, G-rich regions, and R•Y tracts with mirror symmetry are abundant in many genomes and have been implicated in inducing genomic instability, in part through the formation of DNA strand breaks triggered by the transient formation of non-B DNA conformations [14]–[17], [33], [38], [56]–[58]. To identify such structure-forming sequences in mycobacteria, we conducted *in silico* genome-wide searches for repetitive motifs (Table S4) in the reference genomes of *M. smegmatis* and *M. tuberculosis*, the causative agent of tuberculosis, and *E. coli*.

**Figure 2:**
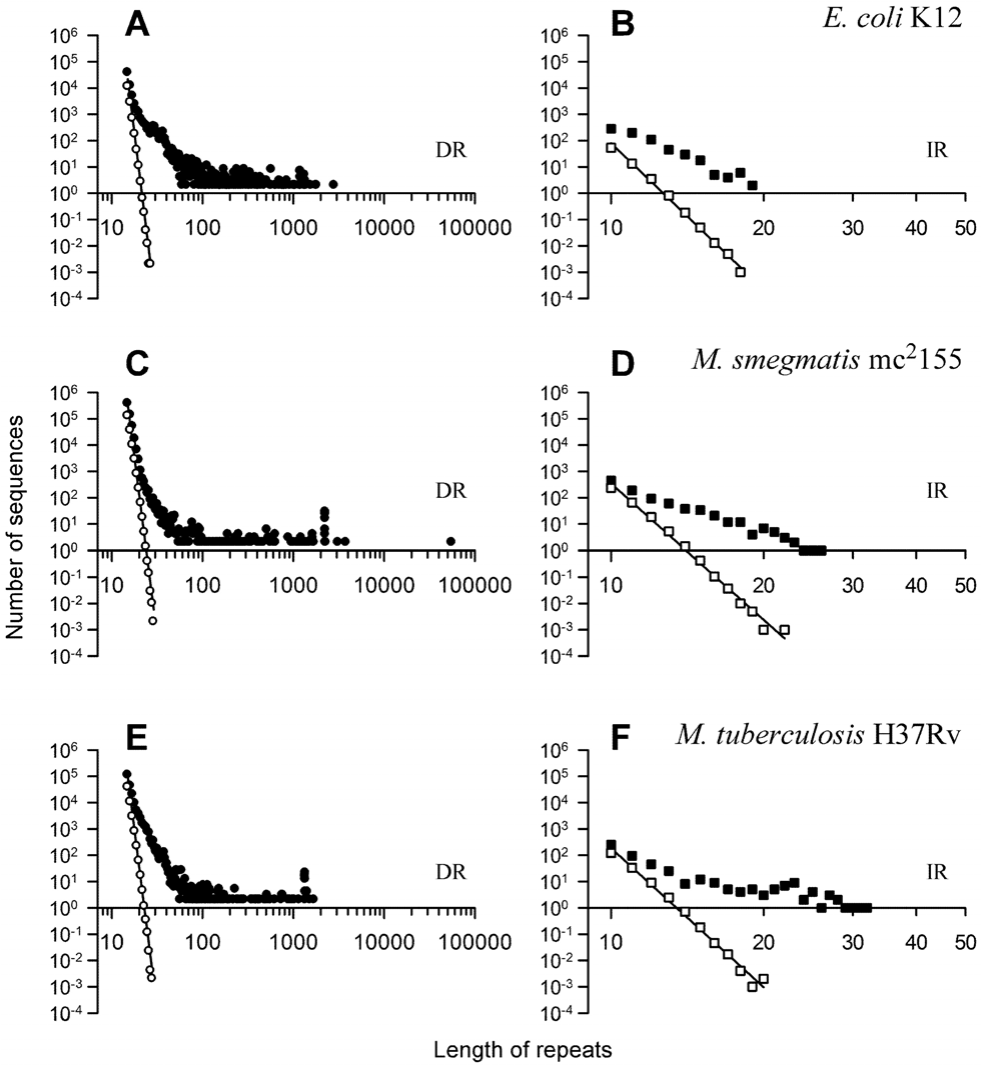
Direct and inverted repeats are overrepresented in mycobacterial and *E. coli* genomes. Genome-wide distributions of DRs and IRs. *Panels A* and *B*, *E. coli* K12; *panels C* and *D*, *M. smegmatis*; *panels E* and *F*, *M. tuberculosis*; *left panels*, direct repeats (*DR*); *right panels*, inverted repeats (*IR*); *x-axes*, length of repeats in nucleotides; *y-axes*, number of repeats; *open symbols*, predicted random distributions; *closed symbols*, observed distributions.

For each repeat type, we assessed if the numbers and lengths of repeats were greater than expected by chance alone. Hence, we compared the *observed* genome-wide distributions with those *predicted* by randomly reshuffling the chromosomal sequences, which generates Gaussian ensembles of repeat numbers as a function of length. Note that for sets of repeats, such as the set of >24 IS1096 elements in the *M. smegmatis* mc^2^155, each element was counted as one repeat. For DRs, the maximum lengths of the predicted sequences did not exceed ∼20–25 bases, whereas the observed length distributions showed the presence of repeats up to thousands of bp in all three genomes, with a gradual decline in the number of sequences as a function of length (Figure 2A, C and E). We found that 1,146,588 nt of DRs (33.58% of the total length of all DRs) accounted for 165 genes annotated to the PE, PE-PGRS, and PPE families in the *M. tuberculosis* genome, whereas only 12,575 nt of DRs (0.13% of the total length of all DRs) in 10 such annotated genes were found in the *M. smegmatis* genome. Thus, the PE and PPE gene families contribute to the distribution of DRs substantially more in *M. tuberculosis* than in *M. smegmatis*. Transposons also contributed to the high number of DRs, particularly in mycobacteria, although this is unlikely to fully account for the differences between mycobacteria and *E. coli*.

IRs were found to be overrepresented in the three bacterial species (Figure 2B, D and F); however, both the difference between the predicted and observed maxima in bp lengths (Figure 2 and Table S5) and the maximum numbers of observed sequences (Figure 2, maximum *y-axis* values) were ∼3 orders of magnitude smaller for IRs than for DRs (maxima in bp lengths near *y* = 10^0^ was ∼14 bp *vs.* ∼19–32 bp, i.e. ∼2-fold difference for IRs and ∼23 bp *vs.* ∼1,697–55,473 bp, i.e. ∼50–2,000-fold difference for DRs; max. *y-axes* = ∼250–460 bp for IRs and ∼40,000–400,000 bp for DRs).

Tandem repeats only accounted for a small proportion (9% in *E. coli*, 4% in *M. tuberculosis*, and 3% in *M. smegmatis*) of all DRs (Table S5), such that ∼60,000 (*E. coli*), ∼190,000 (*M. tuberculosis*) and ∼585,000 (*M. smegmatis*) non-tandem DRs exist in these genomes. Hence, the genome-wide potential for genomic instability due to DR interactions, such as during replication, is ∼3 to 10-fold higher in the two mycobacterial species than in *E. coli*. For G-quadruplex, Z-DNA, and triplex-forming sequences, no differences were observed between the predicted and observed distributions (data not shown). In summary, a significantly greater number of long DRs than expected by chance alone, along with a modest enrichment in IRs, are present in the three bacterial species, particularly in the mycobacterial genomes, which may serve as substrate for genomic instability.

### DRs induce precise deletion of the intervening sequence in mycobacteria but not in *E. coli*

To assess the contribution of DRs and IRs to deletions in mycobacterial genomes, synthetic constructs were engineered and integrated in *M. smegmatis* by L5 site-specific recombination at the *attB* site [59]. Two pairs of IRs (Oligo^IR^) (Figure 1A), each predicted to fold into a cruciform structure comprising a 16 and 18 bp stem with an EcoRI (GAATTC) site in the center (Figure 1B), or a 38-bp DR (Oligo^DR^) (Figure 1), also containing an EcoRI site in the center, were cloned within the *hph* gene so as to abrogate the Hyg^R^ phenotype (Figure 1). We reasoned that repair of DSBs or resumption of arrested replication forks within the IRs or DRs would be assisted by the homologous EcoRI single-stranded loop of cruciforms (Oligo^IR^), or the 38-bp DR sequences, thereby leading to the precise deletion of the cloned inserts and reconstitution of the *hph* gene. The *aacC1* gene conferring Gm^R^ was also cloned in both orientations between the DRs and IRs with the purpose of detecting imprecise deletions that would otherwise not confer Hyg^R^. Oligo^IR^ and Oligo^DR^ were further flanked by the *aph* (Km^R^) and *lac*Z genes to allow for scoring large deletions further away from the repeats through the loss of Km^R^ and blue color (Figure 1A).

**Figure 3:**
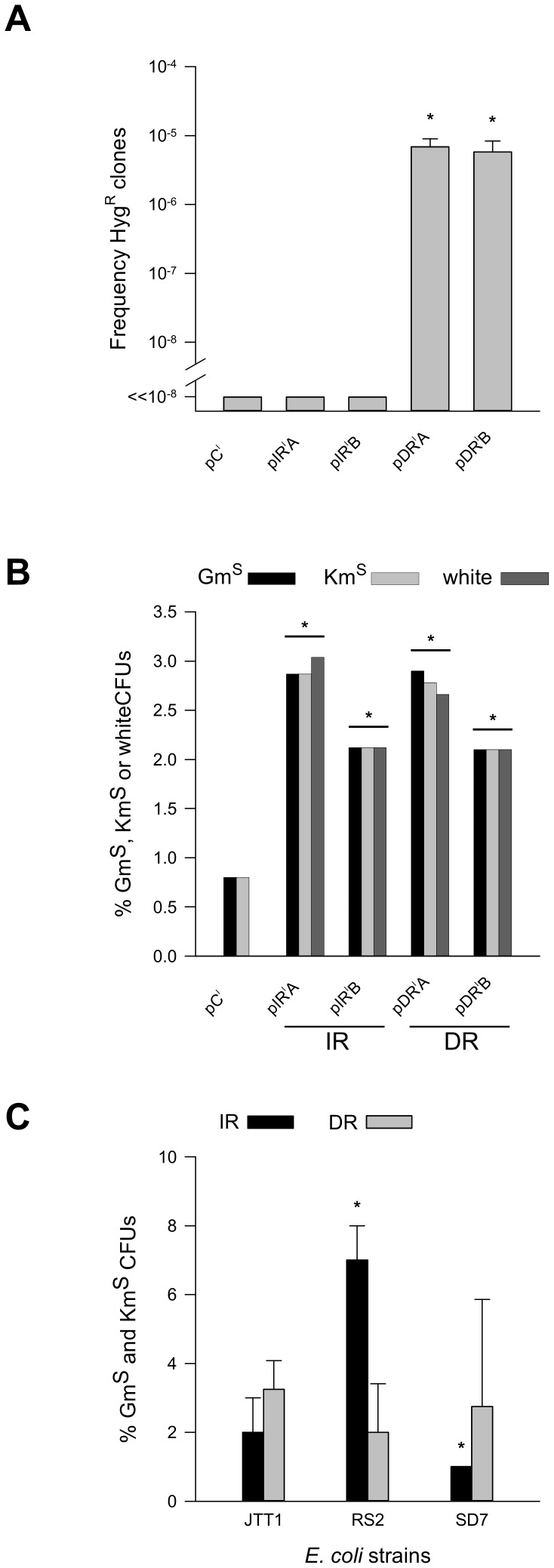
Oligo^DR^ and Oligo^IR^ induce genetic instabilities in *M. smegmatis* and *E. coli*. *Panel A*, reconstitution of *hph* (Hyg^R^) gene through the precise deletion of the *aacC1* (Gm^R^) is only detected with the integrated Oligo^DR^, but not Oligo^IR^, in *M. smegmatis*. *x-axis*, *M. smegmatis* mc^2^155 strains: *pC^I^*, pMV306KmHyg::Gm (control); *pIR^I^A*, pMV306KmHyg::Oligo^IR^Gm^A^ *lacZ*; *pIR^I^B*, pMV306KmHyg::Oligo^IR^Gm^B^ *lacZ*; *pDR^I^A*, pMV306KmHyg::Oligo^DR^Gm^A^ *lacZ*; *pDR^I^B*, pMV306KmHyg::Oligo^DR^Gm^B^ *lacZ*; *y-axis*, frequencies of Hyg^R^ CFUs: *asterisks*, p<0.05 (pair-wise Holm-Sidak test for pIR^I^A, pIR^I^B, pDR^I^A, pDR^I^B against pC^I^). *Panel B*, large deletions occur in both the integrated Oligo^DR^ and Oligo^IR^ constructs in *M. smegmatis*. *x-axis*, as in Panel A; *y-axis*, percent Gm^S^ (*black bars*), Km^S^ (*light grey bars*), and white (*dark grey bars*) CFUs; *IR*, Oligo^IR^; *DR*, Oligo^DR^.; *asterisks*, p<0.05 (χ^2^ test of pIR^I^A, pIR^I^B, pDR^I^A, pDR^I^B against pC^I^, z-test of pIR^I^A against pIR^I^B and pDR^I^A against pDR^I^B). *Panel C*, Oligo^IR^ induces deletions in a supercoiling-dependent manner in *E. coli*. Percent Gm^S^ and Km^S^ CFUs in *E. coli* strains containing various steady-state levels of negative supercoil density. *x-axis*, *E. coli* strains carrying the self-replicating Oligo^IR^ (pIR^E^A) or Oligo^DR^ (pDR^E^A) vectors: *JTT1*, wild-type; *RS2*, topA mutant, higher negative supercoil density than JTT1; *SD2*, topA and gyrB mutant, lower negative supercoil density than JTT1; *y-axis*, percent Gm^S^ and Km^S^ CFUs, geometric mean (+/−SD) from 100 CFUs obtained from 3 independent experiments; *black bars*, Oligo^IR^ (pIR^E^A); *grey bars*, Oligo^DR^ (pDR^E^A); *asterisks*, p<0.05 (χ^2^ test of SD7 against RS2 and RS2 against JTT1).

Selection for Hyg^R^ CFUs indicating on precise deletion within repeated sequences yielded frequencies of 6.9±2.2 and 5.8±2.5×10^−6^ for pDR^I^A and pDR^I^B (superscript I stands for integrated), respectively, which harbored the Oligo^DR^ constructs and the *aacC1* (Gm^R^) gene in either orientation [A (pDR^I^A) or B (pDR^I^B); Figure 3A]. By contrast, no CFUs were obtained out of ∼8.5×10^9^ plated cells when Oligo^IR^ was integrated in pIR^I^A (*aacC1* in orientation A), pIR^I^B (*aacC1* in orientation B) or in the control pC^I^, which is devoid of DR or IR sequences (Figure 3A). Thus, the 38-bp DR in Oligo^DR^ stimulated the rate of deletion of the intervening sequence (precise deletions) by at least 1,000-fold (p = 0,001 for pDR^I^A, p = 0,003 for pDR^I^B), regardless of the orientation of the internal *aacC1* gene. The Hyg^R^ phenotype was confirmed by PCR analyses using *hph*-specific primers (Figure S4 and Table S3).

Next, we investigated whether the Oligo^DR^-stimulated precise deletions were specific to *M. smegmatis*. To address this, the control, direct (Oligo^DR^) and inverted (Oligo^IR^) repeat-containing inserts were transformed in both *E. coli* and *M. smegmatis* using a self-replicating episomal plasmid system (Figure S1 and Table S2). In *M. smegmatis*, both the control (pC^E^; superscript E, episomal) and the Oligo^IR^-containing plasmids (pIR^E^A and pIR^E^B) yielded Hyg^R^ CFUs at frequencies of 2.2±0.3 and 2.4±0.3×10^−7^, respectively (Figure S5). However, the Oligo^DR^-containing plasmids in pDR^E^A and pDR^E^B led to an ∼1,000-fold increase (p = 0,001) in Hyg^R^ CFUs to ∼2.0±0.7 and 2.5±1.0×10^−4^, respectively (Figure S5), in agreement with the data obtained from the integrated system. By contrast, no Hyg^R^ CFUs were rescued in *E. coli* with any of the self-replicating plasmids. Hence, we conclude that 1) the ∼1,000-fold stimulation of precise deletions elicited by the 38-bp DRs was specific to *M. smegmatis*; and 2) both the integrated and episomal plasmid systems were concordant in revealing this type of mutational event.

### IRs and DRs induce large deletions in both *M. smegmatis* and *E. coli*

The lack of Hyg^R^ CFUs in the presence of Oligo^IR^ and the induction of precise deletions by Oligo^DR^ in *M. smegmatis* but not in *E. coli* raised the question as to whether the sequences were able to form the predicted cruciform and looped-out structures, or whether differences in the repair or replication systems between these two species (such as the presence of NHEJ in *M. smegmatis*) were involved. To address these possibilities, *M. smegmatis* carrying the integrated Oligo^IR^ (pIR^I^A and pIR^I^B) or Oligo^DR^ (pDR^I^A and pDR^I^B) constructs were screened for large deletions by the loss of Km^R^ or blue phenotypes caused by the inactivation of the *aph* and *lacZ* genes, respectively (Figure 1). Km^S^ CFUs were detected at approximately 0.8% in the control pC^I^ (Figure 3B). However, a significant 3- to 4-fold increase in Km^S^ and white CFUs was observed in the presence of either Oligo^IR^ or Oligo^DR^ (p = 0,0005 for pIR^I^A, p = 0,01 for pIR^I^B, p = 0,001 for pDR^I^A, p = 0,01 for pDR^I^B), depending on the orientation of the internal *aacC1* (Figure 3B). Specifically, orientation A in pIR^I^A (Oligo^IR^) and pDR^I^A (Oligo^DR^), in which the *hph* and *accC1* genes were transcribed from complementary strands, yielded greater instabilities (p = 0,03) than the alternative orientation B (Figures 1A and 3B). These results support the conclusion that both IRs and DRs were able to induce large deletions in *M. smegmatis*. Moreover, the greater instability observed by divergent transcription of the *aacC1* and *hph* genes (orientation A, Figure 1A) was consistent with our expectation, since this enables the accumulation of greater negative supercoiling during RNA polymerase translocation than same-sense transcription, which should favor B-DNA to non-B DNA structural transitions [60], [61].

To assess whether the IRs and DRs were giving rise to non-B DNA structures, Oligo^IR^ (pIR^E^A and pIR^E^B) and Oligo^DR^ (pDR^E^A and pDR^E^B) were transformed as self-replicating plasmids into isogenic *E. coli* strains that, because of mutations in the gyrase and topoisomerase I genes (Table S1), contained different steady-state levels of negative supercoiling. The use of *E. coli* strains deficient in gyrase/topoisomerases is a convenient and well-documented tool to address the requirement of torsional stress on the transitions between B-DNA and non-B DNA structures [62], [63]. In the wild-type JTT1 strain, in the presence of Oligo^IR^ ∼2±1% CFUs were found to be Gm^S^ and Km^S^ as a result of large deletions (Figure 3C). In the RS2 strain, which sustains higher levels of negative supercoil densities than JTT1, a significant increase (p = 0,02) in Gm^S^ and Km^S^ (7±1%) CFUs was observed, whereas in the SD7 strain, which is characterized by low levels of negative supercoil densities, a significant reduction (p = 0,002) in deletions (as assessed by Gm^S^ and Km^S^ (1±0%) CFUs) compared to RS2 was revealed (Figure 3C). Alternatively, no differences in the fractions of Gm^S^ and Km^S^ CFUs were noted in these strains for the Oligo^DR^-containing plasmids (Figure 3C).

These results are consistent with the conclusion that the IRs in Oligo^IR^ were able to fold into cruciform structures, which then served as substrates for the generation of large deletions, both in *E. coli* and *M. smegmatis*. The lack of induction of large deletions by Oligo^DR^ in *E. coli* suggests that the distant repeats were either prevented from interacting to give rise to slipped structures or, if DNA secondary structures were formed, they were resolved in an error-free manner. Alternatively, differences in DNA replication-dependent misalignment between the repeats might have been involved. In summary, DRs were substantially more mutagenic in *M. smegmatis* than in *E. coli*.

### The precise deletions stimulated by DRs in *M. smegmatis* occur independently of NHEJ, HR and SSA

**Figure 4:**
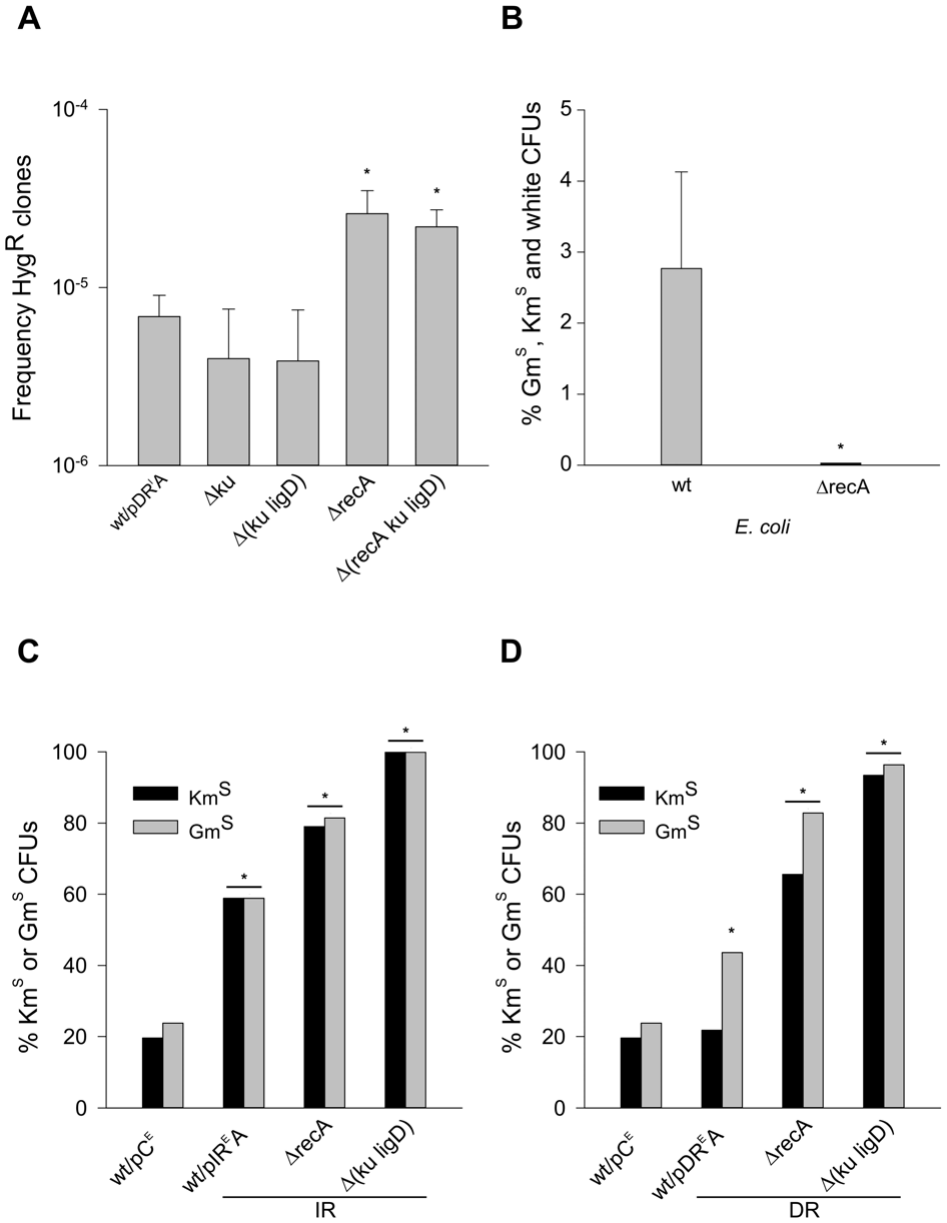
Deletions occur independently of NHEJ and HR in *M. smegmatis*. *Panel A*, precise deletions stimulated by DRs occur independently of NHEJ and are increased in the absence of RecA. *x-axis*, *M. smegmatis* strains: *wt/pDR^I^A*, wild-type/pMV306KmHyg::Oligo^DR^Gm^A^ *lacZ*; *Δku*, Δku/pDR^I^A; *Δ(ku ligD)*, ΔkuΔligD/pDR^I^A; *ΔrecA*, ΔrecA/pDR^I^A; *Δ(recA ku ligD)*, ΔrecAΔkuΔligD/pDR^I^A; *y-axis*, frequencies of Hyg^R^ CFUs; asterisks, p<0.05 (pair-wise Holm-Sidak test for mutant strains against wild-type). *Panel B*, large deletions are stimulated by HR (RecA) in *E. coli*. *x-axis*, *E. coli* strains carrying the self-replicating Oligo^IR^ vector pIR^E^A; *wt*, KMBL1001; *ΔrecA*, Top10 mutant; asterisks, p<0.05 (χ^2^ test of mutant strains against wild-type). *Panels C and D*, large deletions occur independently of NHEJ and HR in *M. smegmatis*. *x axis*, *M. smegmatis* strains; *y-axis*, percent Km^S^ (*black bars*) or Gm^S^ (*grey bars*) CFUs. *Panel C*, *M. smegmatis* strains: *wt/pC^E^*, wild-type/pMV206KmHyg::Gm (control); *wt/pIR^E^A*, wild-type/pMV206KmHyg::Oligo^IR^Gm^A^ *lacZ*; *Δ(ku ligD)*, ΔkuΔligD/pIR^E^A; *ΔrecA*, ΔrecA/pIR^E^A. *Panel D*, *M. smegmatis* strains: *wt/pC^E^*, as in Panel D; *wt/pDR^E^A*, wild-type/pMV306KmHyg::Oligo^DR^Gm^A^ *lacZ*; *Δ(ku ligD)*, ΔkuΔligD/pDR^E^A; *ΔrecA*, ΔrecA/pDR^E^A (asterisks, p<0.05 (χ^2^ test of mutant strains against wild-type).

In addition to HR, a conserved pathway for the repair of DSBs in most organisms, *M. tuberculosis* and *M. smegmatis* (but not *E. coli*) also express an NHEJ activity that uses Ku and DNA ligase D (LigD) to process DSBs [44], [64], [65] without the need for large regions of sequence homology. Thus, we investigated whether the precise deletions induced by the chromosomally integrated Oligo^DR^ in *M. smegmatis* (Figure 3A) involved either the HR or the NHEJ pathways. To this end, we employed mutant strains (Table S1) in which either one or both DNA repair pathways were inactivated [45], [46]. Knocking out the NHEJ pathway in the Δ(ku ligD) *M. smegmatis* mutant did not significantly impair the ability of Oligo^DR^ [pDR^I^A (p = 0,5) and pDR^I^B (p = 0,2)] to stimulate precise deletions, as assessed by the number of Hyg^R^ CFUs (Figure 4A).

**Figure 5:**
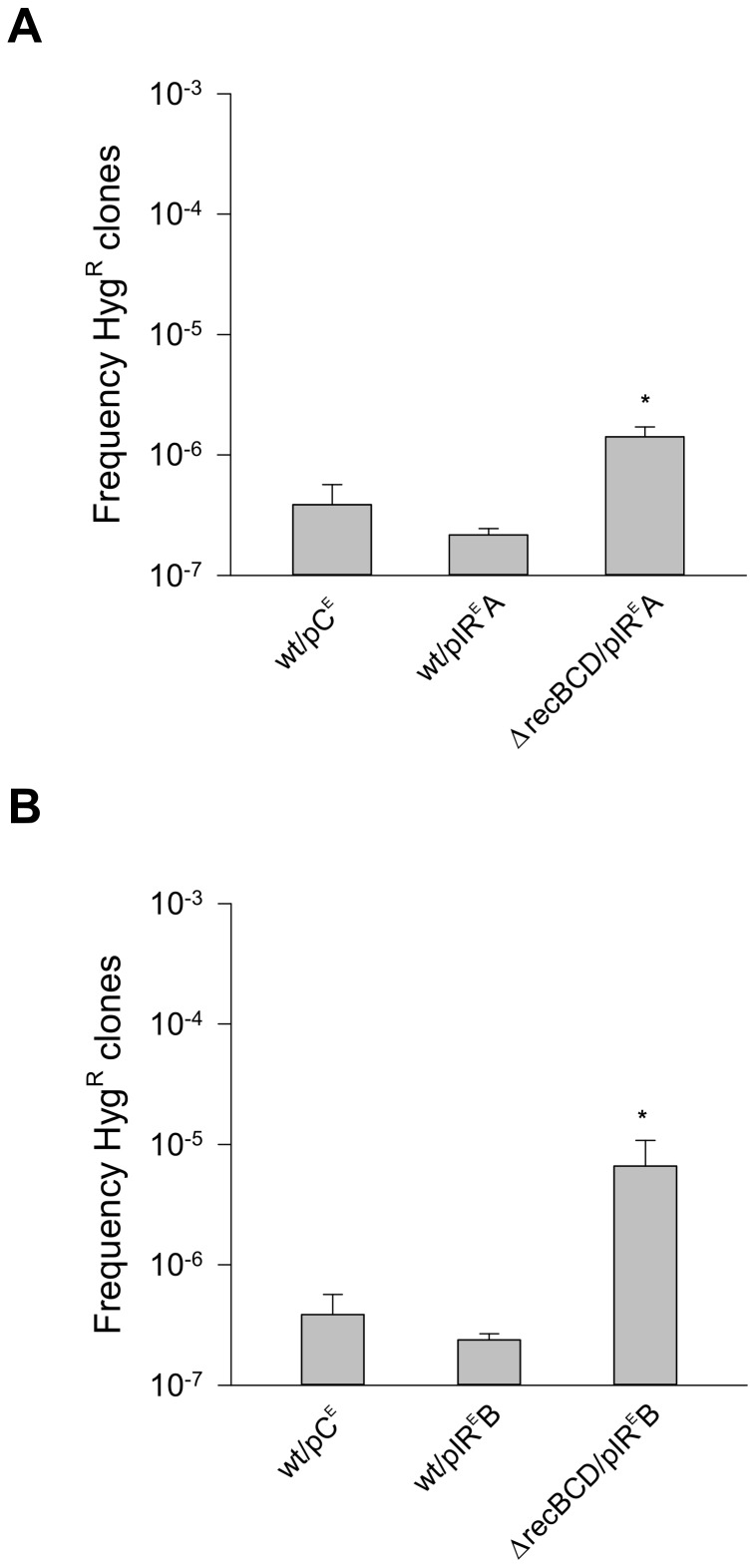
Precise deletions and SSA. RecBCD deficiency increases the frequency of precise deletions between IRs. *Panels A and B*, *x-axis*, *M. smegmatis* strains; *y-axis*, frequencies of Hyg^R^ CFUs; *asterisks*, p<0.05 (pair-wise Holm-Sidak test for ΔrecBCD *M. smegmatis* against wild-type). *Panel A*, *wt/pC^E^*, wild-type/pMV206KmHyg::Gm (control); *wt/pIR^E^A*, wild-type/pMV206KmHyg::Oligo^IR^Gm^A^ *lac*Z; *ΔrecBCD/pIR^E^A*, ΔrecBCD/pMV206KmHyg::Oligo^IR^Gm^A^ *lac*Z. *Panel B*, *wt/pC^E^*, as in Panel A; *wt/pIR^E^B*, wild-type/pMV206KmHyg::Oligo^IR^Gm^B^; *ΔrecBCD/pIR^E^B*, ΔrecBCD/pMV206KmHyg::Oligo^IR^Gm^B^.

RecA is a critical protein that facilitates the strand exchange reaction during HR [66]. In the absence of RecA (Δrec *M. smegmatis*), Oligo^DR^-dependent precise deletions were significantly enhanced (∼5-fold; p = 0,0006 for pDR^I^A, p = 0,00001 for pDR^I^B) relative to the wild-type strain (Figure 4A). This indicates that strand invasion is not required for direct repeat recombination, but in fact may prevent these events. Similar findings have been reported in yeast and mammalian cells [67], [68]. In addition, the ΔrecA-dependent enhancement of precise deletions was not compromised in the absence of NHEJ in the Δ(recA ku ligD) *M. smegmatis* triple mutant. These results indicate that neither HR nor NHEJ were involved in the process(es) that deleted the sequences separating the distant DRs. Recently, a third DNA repair pathway based on single-strand annealing (SSA) has been reported in *M. smegmatis* [49], which complements HR and NHEJ in processing DSBs at short DRs, and which relies on a dedicated RecBCD complex for end-resection. The ΔrecBCD *M. smegmatis* mutant strains (ΔrecBCD/pDR^E^A, *aacC1* in orientation A, ΔrecBCD/pDR^E^B, *aacC1* in orientation B), in which the entire RecBCD operon was ablated, yielded precise deletions at frequencies that were indistinguishable from the wild-type strains (wt/pDR^E^A and wt/pDR^E^B), (Figure S6). This was in contrast with the results obtained with Oligo^IR^ (ΔrecBCD/pIR^E^A and ΔrecBCD/pIR^E^B), in which the fractions of precise deletions increased (p = 0,01) relative to the wild-type strains (wt/pIR^E^A and wt/p IR^E^B) ∼8- to 10-fold (Figure 5). Thus, we conclude that none of the three canonical DSB repair mechanisms, HR, NHEJ and SSA, was required for generating precise deletions between the distant DRs of Oligo^DR^ in *M. smegmatis*. In contrast, RecBCD was involved in preventing precise deletions between the distant pairs of IRs in Oligo^IR^.

### IRs and DRs induced the excision of Oligo^IR^ and Oligo^DR^ by activating L5 site-specific recombination

In an effort to elucidate the potential mechanisms underlying the stimulation of large deletions by DRs and IRs in *M. smegmatis* (Figure 3B), we analyzed the *attB* loci of 118 white and Km^S^ mutant CFUs by PCR, Southern hybridization, and DNA sequencing (Figure S7), including 3 CFUs from the control wt/pC^I^ and 115 CFUs from strains harboring Oligo^DR^ (pDR^I^A and pDR^I^B) or Oligo^IR^ (pIR^I^A and pIR^I^B). These 115 CFUs comprised 31 wild-type, 38 Δku, 30 Δ(ku ligD), and 16 ΔrecA *M. smegmatis* strains. In all 118 cases, the entire integrated plasmid was deleted, whereas the *attB* sequences were precisely reconstituted. Integration of mycobacteriophage L5 is catalyzed primarily by the phage-encoded tyrosine integrase (Int-L5) and the host-encoded protein, mIHF, whereas the excision reaction during the prophage lytic cycle depends upon the recombination directionality factor (RDF), the product of phage gene 36 (Xis-L5) [69]. Our results suggest that DRs and IRs acted as stimulators of mycobacteriophage L5 site-specific recombination.

### HR and NHEJ guard against DNA nucleolytic processing triggered by DR and IR sequences

Because of the stimulation of L5-dependent recombination by the DR and IR sequences, we used Oligo^IR^ (pIR^E^A and pIR^E^B) and Oligo^DR^ (pDR^E^A and pDR^E^B) as self-replicating plasmids, which uncouples the repeats from L5 site-specific recombination, to compare the interaction of DRs and IRs with the host DNA repair systems (*M. smegmatis* and *E. coli*). Thus, we determined the numbers of Gm^S^, Km^S^ and/or white CFUs in wild-type and mutant strains lacking components of HR (*M. smegmatis* and *E. coli*), NHEJ (*M. smegmatis*), or SSA (*M. smegmatis*). In the Top10 *E. coli* mutant strain (Table S1), absence of RecA significantly repressed the instabilities induced by Oligo^IR^ (pIR^I^A and pIR^I^B) ∼10-fold (p = 0,0008 and p = 0,00001, respectively) relative to the wild-type KMBL1001, from 2.8±1.0% to undetectable levels (Figure 4B). This implies that RecA, and thus HR (or other RecA dependent processes, such as the induction of an SOS response and/or the regulation of translesion DNA synthesis), was involved in IR-dependent deletions. The wild-type *M. smegmatis* strain mc^2^155 harboring the control vector (pC^E^, no DR or IR sequences) gave rise to spontaneous Km^S^ or Gm^S^ colonies at frequencies of ∼20% (Figure 4C and D), 30- to 40-fold more frequently than pC^I^ (the corresponding wild-type strain harboring the integrated control vector, Figure 3B), and ∼7-fold higher than in *E. coli* (Figure 4B). A 20% plasmid loss after 48 h culture on nonselective media was expected for this plasmid system, which is based on the pAL5000 origin from *M. fortuitum*. pAL5000-containing vectors are commonly used for growth in *E. coli*, mycobacteria, and for the expression of foreign antigens in recombinant BCG vaccines [70]–[74]. Plasmid DNA was not detectable by DNA isolation or by PCR analysis in white *M. smegmatis* mutant colonies sensitive to Km and Gm, implying that DNA was processed by nucleases and, thus, plasmid was lost. The presence of Oligo^IR^ (pIR^E^A) significantly increased the fractions of Km^S^/Gm^S^ ∼3-fold (p = 0,00001) (Figure 4C), supporting the conclusion that, as in *E. coli* (Figure 3C), cruciform structures were extruded from the IRs and triggered instability through DSBs. Absence of RecA (ΔrecA *M. smegmatis*, Figure 4C) led to a further increase (to ∼67–79%, p = 0,00001) in the fraction of Km^S^/Gm^S^ colonies. Similarly, absence of Ku and LigD in Δ(ku ligD) *M. smegmatis* resulted in loss (p = 0,00001) of the Km/Gm resistance genes in the majority of the bacterial population. Thus, in contrast to *E. coli*, in which HR enhanced IR-induced instabilities, NHEJ and HR displayed a protective role in *M. smegmatis*.

The 38-bp DRs in the self-replicating Oligo^DR^ (pDR^E^A) also stimulated (∼2-fold; p = 0,00001) short deletions that disrupted the *aacC1* (Gm^R^) gene in *M. smegmatis* (Figure 4D); however, no induction of large deletions into the flanking *aph* (Km^R^) gene (and therefore plasmid loss) was observed (Figure 4D). The number of Gm^S^ or Km^S^ CFUs that lost plasmids increased significantly (to 66–93%; p = 0,00001) in the absence of either HR (ΔrecA *M. smegmatis*) or NHEJ (Δ (ku ligD) *M. smegmatis*). We postulate that DSBs, which were left unrepaired in strains defective in RecA or NHEJ proteins in *M. smegmatis*, may have been responsible for the increase in DNA processing and subsequent plasmid loss. In summary, we conclude that both NHEJ and HR served a critical role in repairing DSBs induced by slipped DNA-forming DRs and cruciform-forming IR sequences in *M. smegmatis*.

## Discussion

Herein, we report that the reference genomes of two mycobacterial species, *M. smegmatis* and *M. tuberculosis*, are strongly enriched in DRs and IRs. By using two synthetic constructs harboring DRs and IRs, we addressed whether these repetitive motifs may elicit genomic instability in *M. smegmatis*, a model organism that carries all three (HR, NHEJ and SSA) DSB repair pathways. We also used *E. coli* strains, since these enable a direct relationship between repeat DNA-induced genetic instability and negative supercoiling, a topological state known to favor B-DNA to non-B-DNA structural transitions [62], [63], [75]. *E. coli* and *M. smegmatis* represent quite different model systems with regard to DNA metabolism and DNA repair mechanisms. Specifically, *M. smegmatis*, as well as other mycobacteria, are devoid of genes encoding the highly conserved mismatch repair (MMR) proteins MutS and MutL, suggesting a lack in postreplicative MMR [1], [2], [76], [77], whereas *E. coli* is devoid of genes encoding the conserved two-component core NHEJ enzymes, Ku and LigD, leaving the processing of incompatible DSBs to alternative mechanisms [78]–[80].

We report four findings. First, the 38-bp DRs in Oligo^DR^ strongly induced the precise deletion of their ∼1 kb intervening sequence even in the absence of functional DSB repair in *M. smegmatis*. Whereas RecA-independent slipped mispairing between tandem repeats is a well-recognized mechanism leading to replication errors [12], [81], the ability of the *M. smegmatis* replication apparatus to relocate at distant DRs unassisted by canonical DSB repair proteins has not been reported, to our knowledge. Absence of RecA, both alone or in combination with NHEJ deficiency, favored this process, suggesting that either HR prevented deletions or that binding of RecA to single-stranded DNA (either because of looping-out following DR interactions or because of arrested replication forks [82]), inhibited primer relocation. In contrast to Oligo^DR^, precise deletions between the two IRs in Oligo^IR^ were considerably less abundant, being detected only upon loss of the RecBCD operon.

The only mechanism through which *hph* gene reconstitution could have occurred in Oligo^IR^ was by precisely rejoining the two EcoRI sites that separated the two halves of the gene (Figure 1). Based on the *E. coli* mutant data (Figure 3C), in which Oligo^IR^-induced instabilities increased in a supercoiling-dependent manner, we speculate that both IRs were able to fold into cruciform structures; these then underwent strand breaks at the single-stranded EcoRI sequences, which were repaired by microhomology-mediated end-fusion between the two IRs. Because this type of event was stimulated in the absence of RecBCD, we propose that EcoRI-specific DSBs were processed through the SSA pathway in the wild-type strains and by the NHEJ machinery in the ΔSSA strains [41]. In *E. coli*, the absence of precise deletions in IR-containing constructs is consistent with the lack of canonical NHEJ. Thus, SSA would appear to compete effectively with NHEJ in processing repeat-induced strand-breaks in mycobacteria.

Second, both DRs and IRs incited int-L5 site-specific recombination in *M. smegmatis*. The instability of integrated constructs by L5 integrase activity has been reported [83]; we document a strong stimulation of this process by DRs and IRs. In *M. smegmatis*, the directionality of mycobacteriophage L5 integration and excision reactions is governed by the phage recombination directionality factor (RDF) Xis-L5, a small 56 amino acid helix-turn-helix peptide that bends Xis-L5 DNA recognition targets in proximity to *attR* [69]. Xis-L5 does not participate directly in the excision reaction, but instead acts as an architectural protein. We speculate that, if a role for Xis-L5 were to stabilize *attR* and *attL* site interaction leading to synapsis, then such interaction may be facilitated by flexible hinges along the DNA chain at local non-B DNA conformations, such as those formed by slipped and cruciform structures at DRs and IRs, respectively. Other mechanisms are possible, including bidirectional DNA nuclease activity from the DNA secondary structures into the site-specific recombination sites. Regardless of the mechanism involved, it is clear that DR and IR sequences strongly stimulate L5 site-specific recombination.

Third, absence of HR and NHEJ, (ΔNHEJ>ΔHR), resulted in extensive DNA processing and plasmid loss in *M. smegmatis*, indicating that both pathways play important roles in protecting genome integrity from the destabilizing effects of DRs and IRs. In this regard, a contrasting effect was observed between *M. smegmatis* and *E. coli*, since in the latter abrogation of the HR pathway in the ΔrecA strains led to greater genomic stability. The reasons for these differences remain to be clarified.

Fourth, DRs are the most abundant type of repetitive motif in the *M. smegmatis* and *M. tuberculosis* genomes. Short repetitive motifs are a hallmark of insertion sequence (IS) integration sites in mycobacteria. Duplications, deletions and genome shuffling play major roles in driving genomic diversity and evolution, and mycobacterial IS elements, such as IS*1096* and IS*6110*, are believed to contribute to such rearrangements [84], [85]. We found 165 genes annotated to PE, PE-PGRS and PPE families in *M. tuberculosis*, for a maximum contribution to the total length of DRs of 8.2%. In *M. smegmatis*, only 10 such genes were found. The PE and PPE families of proteins confer pathogenic advantage to *M. tuberculosis*, and antigenic variation may result in part from the interactions between the PE/PPE proteins and the emergence of new or rare MHC alleles in the host genome [86]. Thus, it is possible that duplication of these gene families might have been exploited, and therefore maintained, through positive selection as a means to increase diversity through recombination, and perhaps avoid gene decay during evolutionary time in *M. tuberculosis*. A second class of genetic elements thought to generate mosaicism in mycobacteria is represented by mycobacteriophages; at least sixty-two different genomes of these obligate parasites known to infect *M. smegmatis* mc^2^155 have been sequenced [87]. Short repetitive motifs in the bacterial host, such as the 36 DR integration hotspot [88], represent common sites for the integration and excision of such elements, and both mycobacterial DNA repair proteins, such as LigD and Ku [89], and phage integrases may cooperate to carry out the insertion and excision reactions [90]. Mycobacteriophages are characterized by a substantial degree of mosaicism, including the occasional acquisition of mycobacterial sequences, such as the *MetE* gene in the Giles genome [91], [92]. Such mosaicism is thought to arise in part through lateral gene transfer (LGT). A comparison between the *M. tuberculosis* genome and its relatives *M. marinum* and *M. avium* subsp. *paratuberculosis* suggests that at least 80 distinct regions have been acquired by *M. tuberculosis* through LGT, along with genome downsizing, in the course of its adaptation from an environmental ancestor to a specialized human pathogen [93]. Our results raise the possibility that the abundant repetitive elements in mycobacteria might instigate recombination events between the host and mycobacteriophage genomes, contributing to LGT. In summary, we demonstrate that DR and IR sequences lead to genomic instability in *M. smegmatis* by eliciting a diverse repertoire of cellular responses, including interactions with DNA repair pathways (HR, NHEJ and SSA) and the mycobacteriphage L5 site-specific recombination apparatus. Thus, these results provide support for a much broader range of cellular responses to repetitive DNA sequences and their putative secondary structures than previously anticipated, and suggest future studies aimed at addressing the evolutionary aspects and origins of repetitive sequences.

## Supporting Information

Figure S1 **Cloning strategy.** The *hph* gene (Hyg^R^) in the pMV206Km and pMV306Km vectors was disrupted by introducing synthetic oligonucleotides, comprising either two pairs of inverted repeats (IRs) or two direct repeat (DR) units, within an internal EcoRI site of the gene. DRs and IRs were further separated by cloning the *aacC1* (Gm^R^) gene in orientations A and B. This resulted in two pairs of IRs and one pair of DRs, on either side of the *aacC1*. The disrupted *hph* was also flanked, 5′ and 3′ respectively, by the *aph* (Km^R^) and *lac*Z (blue color) genes. *Pag85*, promoter for the *lacZ* gene.
(TIF)

Figure S2 **Construction of** ***M. smegmatis*** **ΔrecBCD strain by replacement of the wild-type recBCD operon with a mutant sequence.** *Panel A*, the chromosomal location of *recBCD* is represented by arrows. The restriction sites (BamHI and ClaI) used for cleaving chromosomal DNA are shown. The restriction DNA fragment and the size of the internal deletion in the mutated copies are shown by thin black arrows. The probe used for Southern blot hybridization is displayed by a grey rectangle. *Panel B*, Southern blot hybridization. *Lane 1*, *M. smegmatis* mc^2^155; *lane 2*, *M. smegmatis* recBCD and ΔrecBCD; *lanes 3, 4, M. smegmatis* ΔrecBCD. *Arrows*, DNA restriction fragments expected for the *M. smegmatis* wild-type recBCD (982 bp) and ΔrecBCD (1601 bp) strains.
(TIF)

Figure S3 **Protocol for assessing genetic instabilities caused by the Oligo^DR^ and Oligo^IR^ sequences in** ***E. coli*** **and** ***M. smegmatis*** **.** *From the top*, competent cells were transformed with Oligo^DR^ (pDR^I^A, pDR^I^B, pDR^E^A, pDR^E^B) or Oligo^IR^ (pIR^I^A, pIR^I^B, pIR^E^A, pIR^E^B) vectors and plated on LB (*E. coli*) or NB (*M. smegmatis*) media containing gentamycin (Gm), kanamycin (Km) and X-gal. CFUs were harvested, plated on non-selective media and grown for 24 h (*E. coli*) or 48 h (*M. smegmatis*) to select for mutants. Cells were then washed and plated at the appropriate dilutions on LB/NB agar plates supplemented with either 5-bromo-4-chloro-3-indoxyl-beta-D-galactopyranoside (X-gal) or hygromycin (Hyg). Colonies from X-gal plates were transferred by replica plating on four LB/NB agar plates, as follows: 1) Km plus Hyg, 2) Km plus Gm, 3) Km and 4) X-gal.
(TIF)

Figure S4 **Oligo^DR^ induces precise deletions between DRs that reconstitute an intact** ***hph*** **gene (Hyg^R^).** Agarose gel electrophoresis of PCR products using *hph* specific primers on DNA isolated from Hyg^R^ colonies. *Lane 1*, 1-kb DNA ladder; *lane 2*, *M. smegmatis* carrying pDR^I^A (Hyg^S^, Gm^R^); *lanes 3–12*, selected Hyg^R^ and Gm^S^ *M. smegmatis* mutant CFUs harboring pDR^I^A or pDR^I^B.
(TIF)

Figure S5 **Reconstitution of** ***hph*** **(Hyg^R^) within the self-replicating Oligo^DR^ and Oligo^IR^ in** ***M. smegmatis*** **.** *x-axis*, *M. smegmatis mc^2^155* strains: *pC^E^*, pMV206KmHyg::Gm (control); *pIR^E^A*, pMV206KmHyg::Oligo^IR^Gm^A^ *lacZ*; *pIR^E^B*, pMV206KmHyg::Oligo^IR^Gm^B^ *lacZ*; *pDR^E^A*, pMV206KmHyg::Oligo^DR^Gm^A^ *lacZ*; *pDR^E^B*, pMV206KmHyg::Oligo^DR^Gm^B^; *y-axis*, frequencies of Hyg^R^ CFUs; *asterisks*, P<0.05 (pair-wise Holm-Sidak test of pIR^E^A, pIR^E^B, pDR^E^A, pDR^E^B against pC^E^).
(TIF)

Figure S6 **Precise deletions and SSA.** *M. smegmatis* ΔRecBCD strains process precise deletions between DRs with similar efficiencies as the wild-type strain. *Panel A and B*, *x-axis*, *M. smegmatis* strains; *y-axis*, frequencies of Hyg^R^ CFUs. *Panel A*, *x-axis*, *wt/pC^E^*, wild-type/pMV206KmHyg::Gm (control); *wt/pDR^E^A*, wild-type/pMV206KmHyg::Oligo^DR^Gm^A^ *lac*Z; *ΔrecBCD/pDR^E^A*, ΔrecBCD/pMV206KmHyg::Oligo^DR^Gm^A^ *lac*Z. *Panel B*, *x-axis*, *wt/pC^E^*, as in Panel A; *wt/pDR^E^B*, wild-type/pMV206KmHyg::Oligo^DR^Gm^B^ *lac*Z; *ΔrecBCD/pDR^E^B*, ΔrecBCD/pMV206KmHyg::Oligo^DR^Gm^B^ *lac*Z.
(TIF)

Figure S7 **The bacteriophage L5 integrase excises the DR- and IR-containing constructs integrated at the** ***attB*** **sites.** Analysis of the *attB* region of selected mutants using Southern blot hybridization. *Panel A*, scheme of the plasmids integrated at the *attB* site of *M. smegmatis* mc^2^155. *SalI*, restriction sites used for DNA cleavage showing the 1,883 bp SalI fragment detectable by the Southern blot hybridization probe in the wild-type strain and the DNA fragments (*grey arrows*) generated by SalI cleavage in the integrated plasmid. *Panel B*, Southern blot hybridization. *Lane 1*, *M. smegmatis* mc^2^155; *lane 2*, *M. smegmatis* mc^2^155/pDR^I^A (pMV306KmHyg::Oligo^DR^Gm^A^ *lacZ*); *lanes 3, 4, 6, 9–17*, Km^S^, Gm^S^ and white mutant CFUs from *M. smegmatis* mc^2^155/pDR^I^A; *lanes 5, 7–8*, Km^R^, Gm^S^ and blue mutant CFUs from *M. smegmatis* carrying pDR^I^A. *Red arrows*, DNA restriction fragments expected for the wild-type strain (1,883 bp) and for *M. smegmatis* mc^2^155/pDR^I^A (4,698 bp and 1,185 bp).
(TIF)

Table S1 Bacterial strains, sources and genetic features.
(RTF)

Table S2 List of plasmids, general description, genetic markers and sources/references.
(RTF)

Table S3 Primer sequences used for PCR amplification.
(RTF)

Table S4 Criteria used for *in silico* analyses of repetitive elements in bacterial genomes.
(RTF)

Table S5 Statistics of *in silico* searches of repeats genome-wide.
(RTF)
