# Supplementary figures and images for: Direct and Inverted Repeats Elicit Genetic Instability by Both Exploiting and Eluding DNA Double-Strand Break Repair Systems in Mycobacteria
Source: PLoS One. 2012 Dec 10;7(12):e51064. doi: 10.1371/journal.pone.0051064 (PMC3519483; doi:10.1371/journal.pone.0051064)

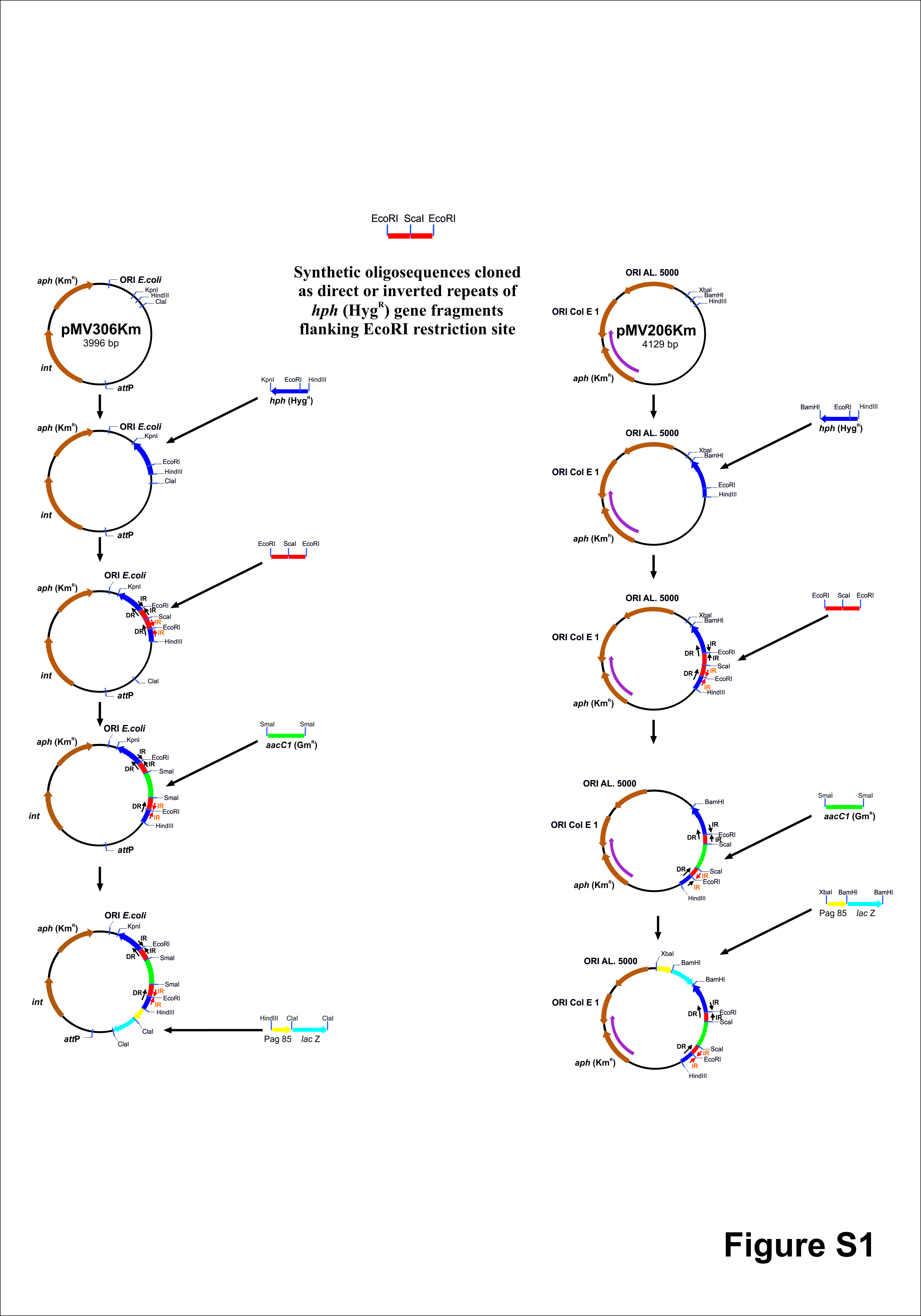

Supplement: Figure S1 — Cloning strategy. The hph gene (HygR) in the pMV206Km and pMV306Km vectors was disrupted by introducing synthetic oligonucleotides, comprising either two pairs of inverted repeats (IRs) or two direct repeat (DR) units, within an internal EcoRI site of the gene. DRs and IRs were further separated by cloning the aacC1 (GmR) gene in orientations A and B. This resulted in two pairs of IRs and one pair of DRs, on either side of the aacC1. The disrupted hph was also flanked, 5′ and 3′ respectively, by the aph (KmR) and lacZ (blue color) genes. Pag85, promoter for the lacZ gene. (TIF) [file pone.0051064.s001.tif]

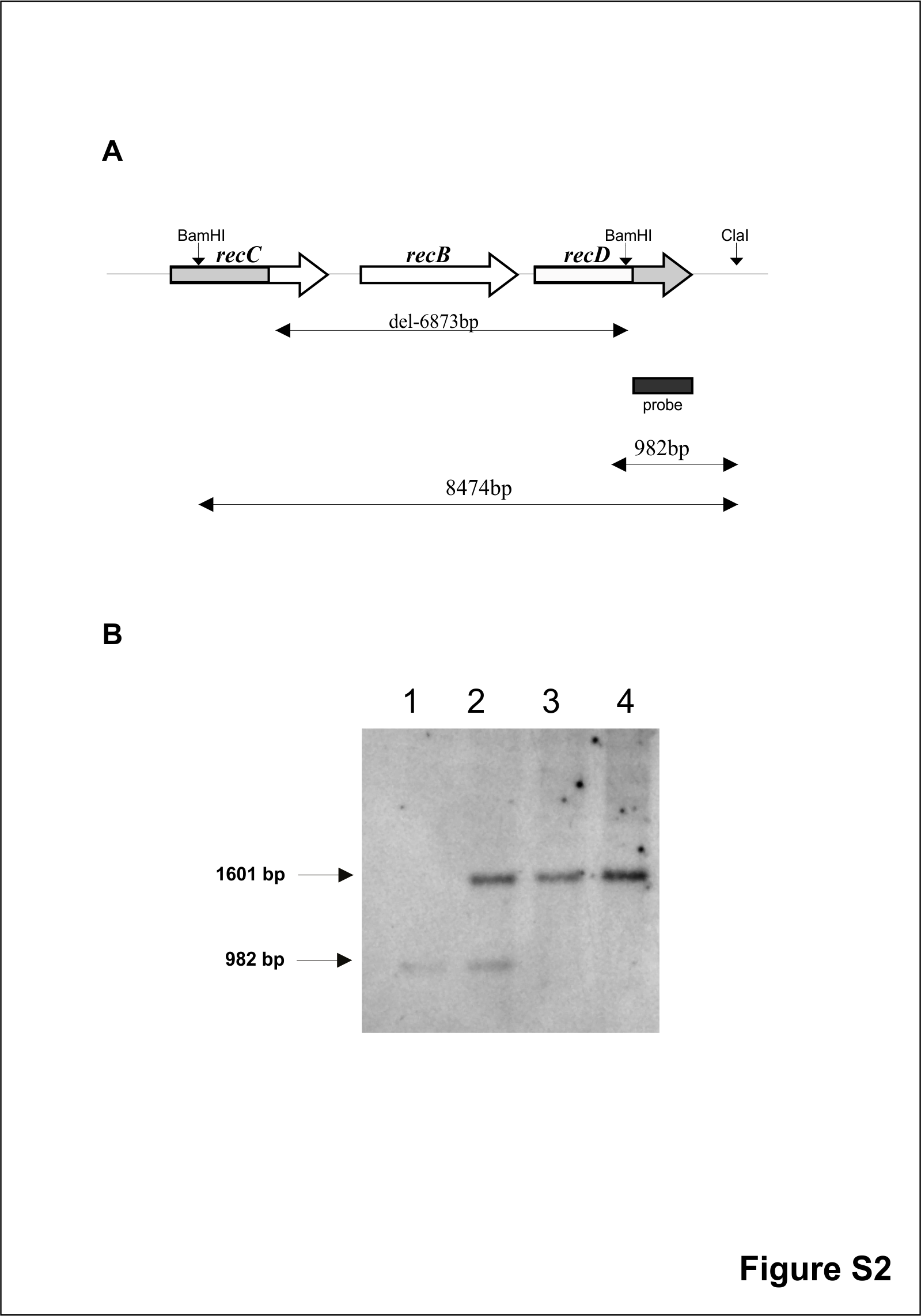

Supplement: Figure S2 — Construction of M. smegmatis ΔrecBCD strain by replacement of the wild-type recBCD operon with a mutant sequence. Panel A, the chromosomal location of recBCD is represented by arrows. The restriction sites (BamHI and ClaI) used for cleaving chromosomal DNA are shown. The restriction DNA fragment and the size of the internal deletion in the mutated copies are shown by thin black arrows. The probe used for Southern blot hybridization is displayed by a grey rectangle. Panel B, Southern blot hybridization. Lane 1, M. smegmatis mc2155; lane 2, M. smegmatis recBCD and ΔrecBCD; lanes 3, 4, M. smegmatis ΔrecBCD. Arrows, DNA restriction fragments expected for the M. smegmatis wild-type recBCD (982 bp) and ΔrecBCD (1601 bp) strains. (TIF) [file pone.0051064.s002.tif]

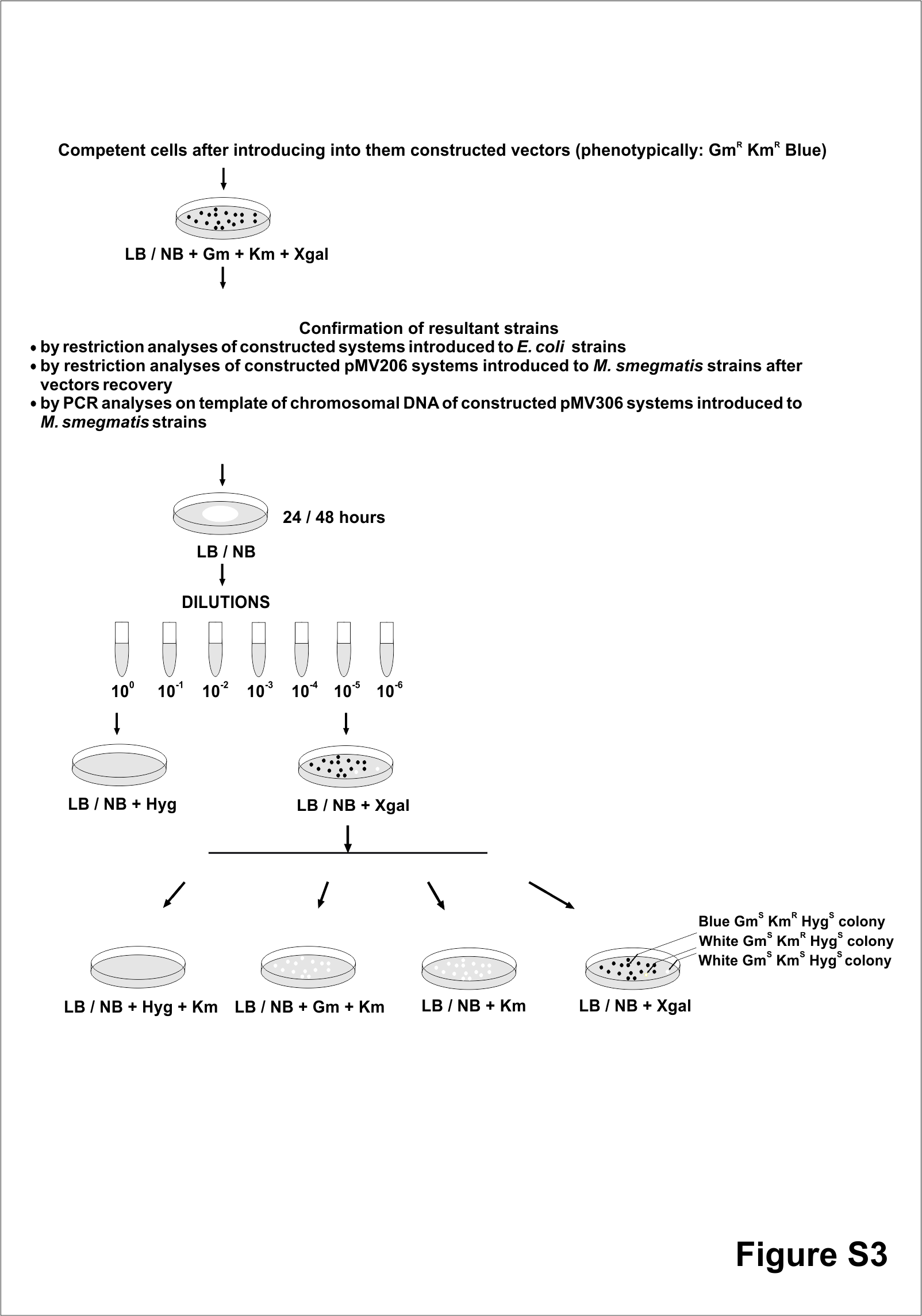

Supplement: Figure S3 — Protocol for assessing genetic instabilities caused by the OligoDR and OligoIR sequences in E. coli and M. smegmatis . From the top, competent cells were transformed with OligoDR (pDRIA, pDRIB, pDREA, pDREB) or OligoIR (pIRIA, pIRIB, pIREA, pIREB) vectors and plated on LB (E. coli) or NB (M. smegmatis) media containing gentamycin (Gm), kanamycin (Km) and X-gal. CFUs were harvested, plated on non-selective media and grown for 24 h (E. coli) or 48 h (M. smegmatis) to select for mutants. Cells were then washed and plated at the appropriate dilutions on LB/NB agar plates supplemented with either 5-bromo-4-chloro-3-indoxyl-beta-D-galactopyranoside (X-gal) or hygromycin (Hyg). Colonies from X-gal plates were transferred by replica plating on four LB/NB agar plates, as follows: 1) Km plus Hyg, 2) Km plus Gm, 3) Km and 4) X-gal. (TIF) [file pone.0051064.s003.tif]

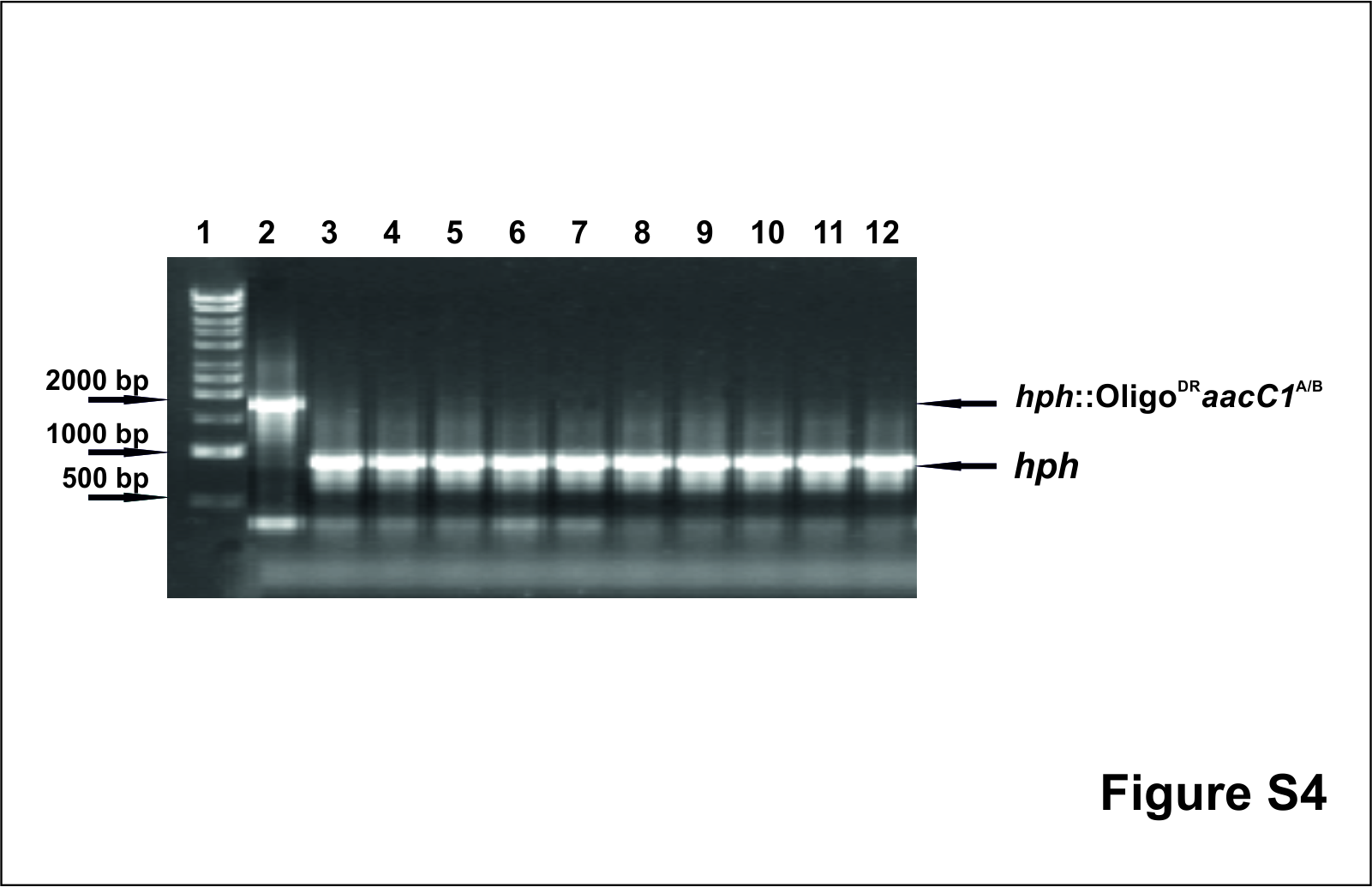

Supplement: Figure S4 — OligoDR induces precise deletions between DRs that reconstitute an intact hph gene (HygR). Agarose gel electrophoresis of PCR products using hph specific primers on DNA isolated from HygR colonies. Lane 1, 1-kb DNA ladder; lane 2, M. smegmatis carrying pDRIA (HygS, GmR); lanes 3–12, selected HygR and GmS M. smegmatis mutant CFUs harboring pDRIA or pDRIB. (TIF) [file pone.0051064.s004.tif]

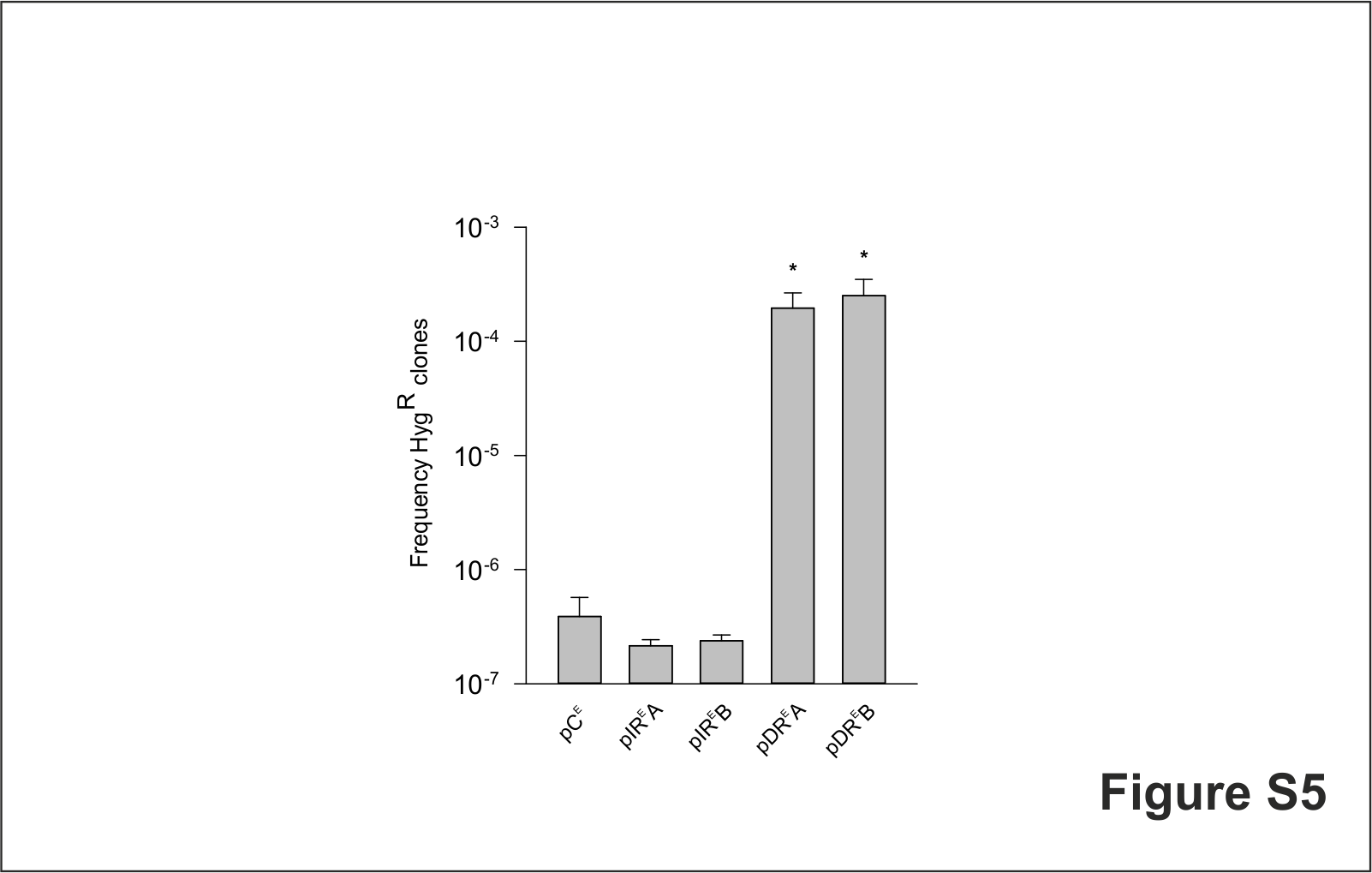

Supplement: Figure S5 — Reconstitution of hph (HygR) within the self-replicating OligoDR and OligoIR in M. smegmatis . x-axis, M. smegmatis mc2155 strains: pCE, pMV206KmHyg::Gm (control); pIREA, pMV206KmHyg::OligoIRGmA lacZ; pIREB, pMV206KmHyg::OligoIRGmB lacZ; pDREA, pMV206KmHyg::OligoDRGmA lacZ; pDREB, pMV206KmHyg::OligoDRGmB; y-axis, frequencies of HygR CFUs; asterisks, P<0.05 (pair-wise Holm-Sidak test of pIREA, pIREB, pDREA, pDREB against pCE). (TIF) [file pone.0051064.s005.tif]

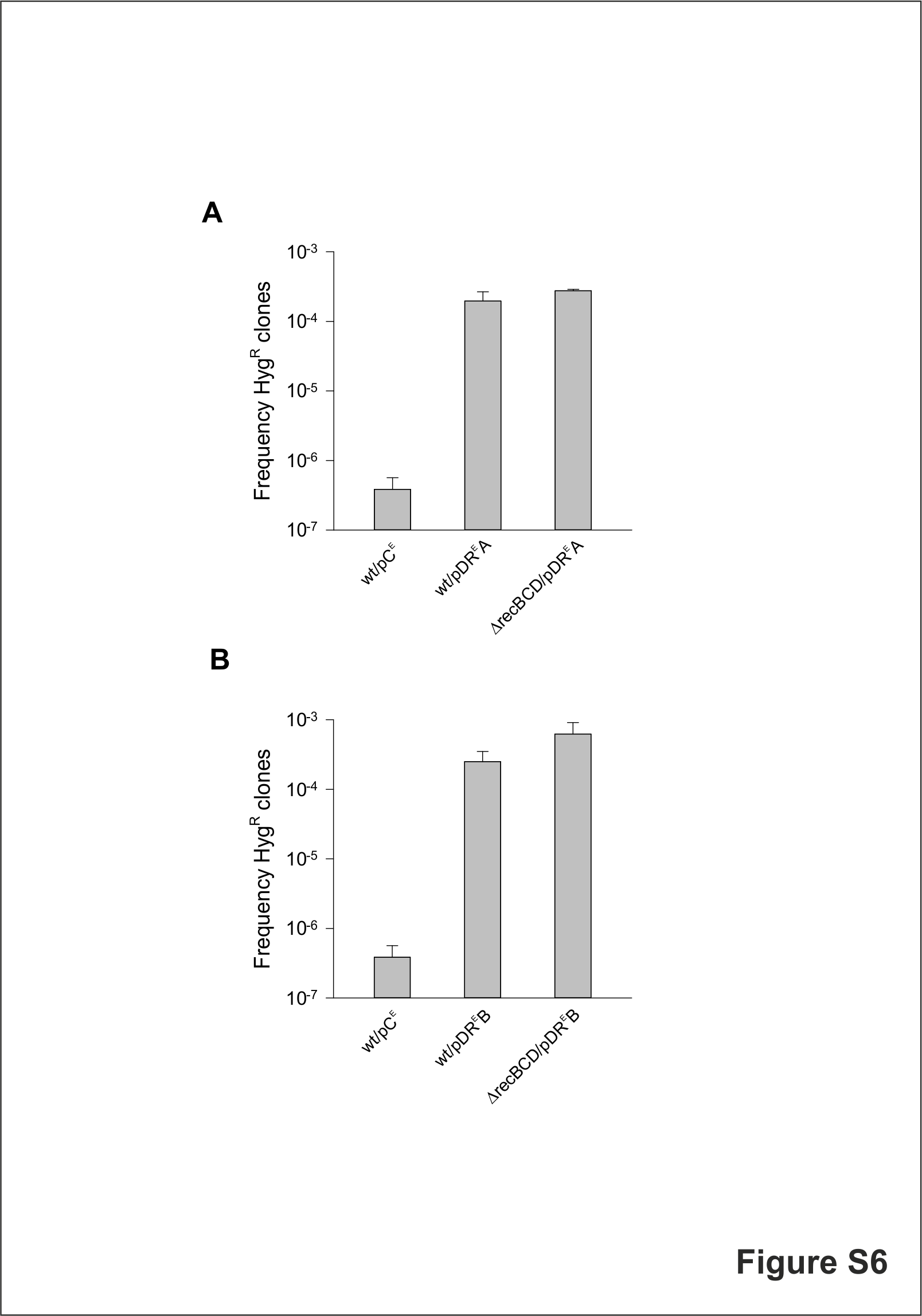

Supplement: Figure S6 — Precise deletions and SSA. M. smegmatis ΔRecBCD strains process precise deletions between DRs with similar efficiencies as the wild-type strain. Panel A and B, x-axis, M. smegmatis strains; y-axis, frequencies of HygR CFUs. Panel A, x-axis, wt/pCE, wild-type/pMV206KmHyg::Gm (control); wt/pDREA, wild-type/pMV206KmHyg::OligoDRGmA lacZ; ΔrecBCD/pDREA, ΔrecBCD/pMV206KmHyg::OligoDRGmA lacZ. Panel B, x-axis, wt/pCE, as in Panel A; wt/pDREB, wild-type/pMV206KmHyg::OligoDRGmB lacZ; ΔrecBCD/pDREB, ΔrecBCD/pMV206KmHyg::OligoDRGmB lacZ. (TIF) [file pone.0051064.s006.tif]

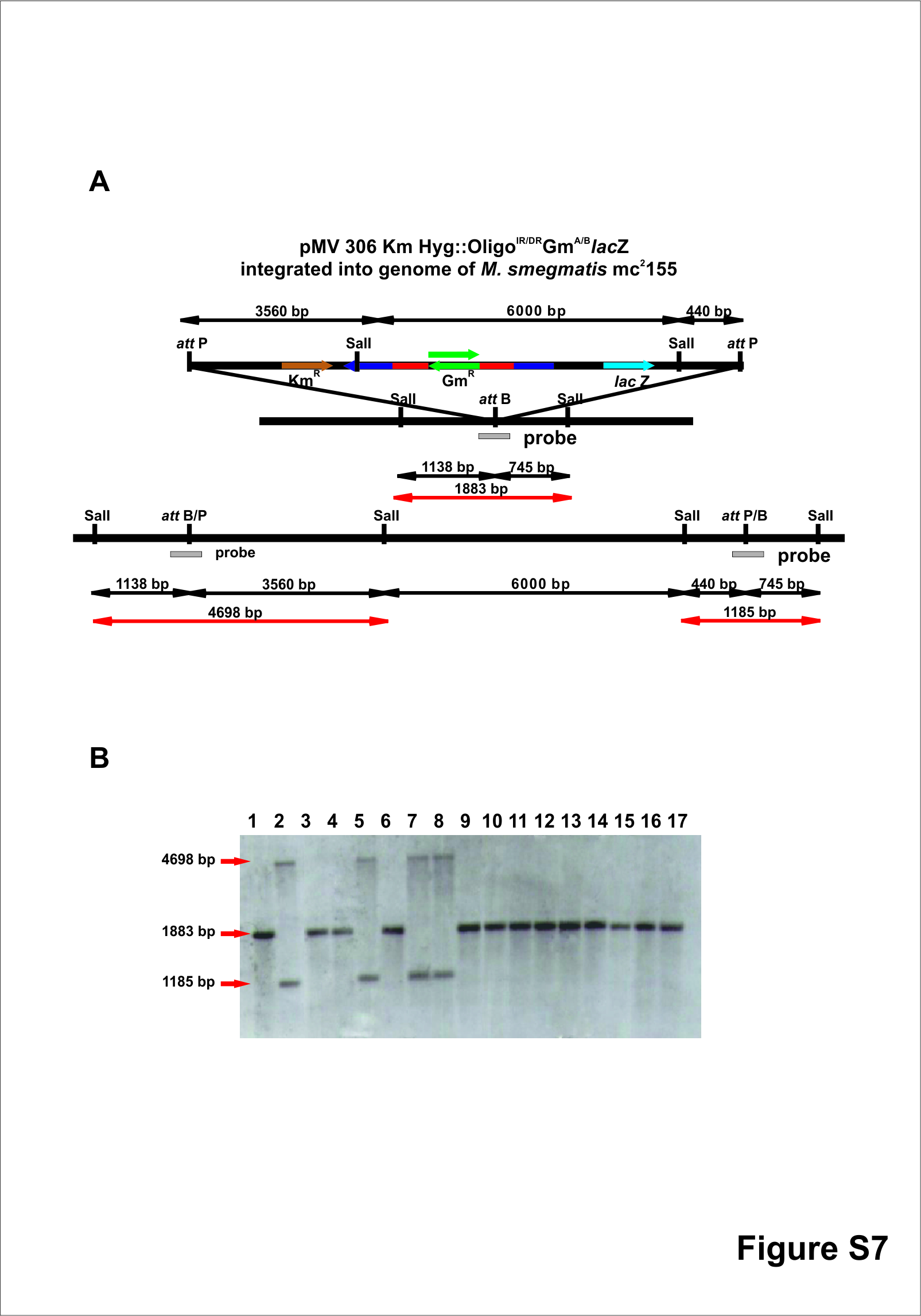

Supplement: Figure S7 — The bacteriophage L5 integrase excises the DR- and IR-containing constructs integrated at the attB sites. Analysis of the attB region of selected mutants using Southern blot hybridization. Panel A, scheme of the plasmids integrated at the attB site of M. smegmatis mc2155. SalI, restriction sites used for DNA cleavage showing the 1,883 bp SalI fragment detectable by the Southern blot hybridization probe in the wild-type strain and the DNA fragments (grey arrows) generated by SalI cleavage in the integrated plasmid. Panel B, Southern blot hybridization. Lane 1, M. smegmatis mc2155; lane 2, M. smegmatis mc2155/pDRIA (pMV306KmHyg::OligoDRGmA lacZ); lanes 3, 4, 6, 9–17, KmS, GmS and white mutant CFUs from M. smegmatis mc2155/pDRIA; lanes 5, 7–8, KmR, GmS and blue mutant CFUs from M. smegmatis carrying pDRIA. Red arrows, DNA restriction fragments expected for the wild-type strain (1,883 bp) and for M. smegmatis mc2155/pDRIA (4,698 bp and 1,185 bp). (TIF) [file pone.0051064.s007.tif]
