# Supplementary material for: Direct and Inverted Repeats Elicit Genetic Instability by Both Exploiting and Eluding DNA Double-Strand Break Repair Systems in Mycobacteria
Source: PLoS One. 2012 Dec 10;7(12):e51064. doi: 10.1371/journal.pone.0051064 (PMC3519483; doi:10.1371/journal.pone.0051064)
Supplement: Table S1 — Bacterial strains, sources and genetic features. (RTF) [file pone.0051064.s008.rtf]

Table S1. Bacterial strains, sources and genetic features.
Species	Strain	Source	Genetic features	
Escherichia coli	Top10	Invitrogen	RecA deficient	
Escherichia coli	KMBL1001	[1]	wt for RecA	
Escherichia coli	JTT1	[2]	wt for negative supercoil density, -ó	
Escherichia coli	RS2		TopA mutation; higher1 -ó than wt	
Escherichia coli	SD7		TopA and GyrB mutations, lower1 -ó than wt	
Mycobacterium smegmatis	mc2155	[3]	wt	
Mycobacterium smegmatis	Ä(ku ligD)	[4]	Ku and LigD deficient	
Mycobacterium smegmatis	ÄrecA		RecA deficient	
Mycobacterium smegmatis	Ä(recA ku ligD)		Ku, LigD and RecA deficient	
Mycobacterium smegmatis	ÄrecBCD	This study	RecBCD deficient	

1 A higher negative supercoil density is referred to as a more negative value of -ó, and a lower negative supercoil density as a less negative value of -ó.


References
1. Moolenaar GF, Moorman C, Goosen N (2000) Role of the Escherichia coli nucleotide excision repair proteins in DNA replication. J Bacteriol 182: 5706-5714.
2. DiNardo S, Voelkel KA, Sternglanz R, Reynolds AE, Wright A (1982) Escherichia coli DNA topoisomerase I mutants have compensatory mutations in DNA gyrase genes. Cell 31: 43-51.
3. Snapper SB, Melton RE, Mustafa S, Kieser T, Jacobs WR, Jr. (1990) Isolation and characterization of efficient plasmid transformation mutants of Mycobacterium smegmatis. Mol Microbiol 4: 1911-1919.
4. Korycka-Machala M, Brzostek A, Rozalska S, Rumijowska-Galewicz A, Dziedzic R, et al. (2006) Distinct DNA repair pathways involving RecA and nonhomologous end joining in Mycobacterium smegmatis. FEMS Microbiol Lett 258: 83-91.
