# Supplementary material for: Direct and Inverted Repeats Elicit Genetic Instability by Both Exploiting and Eluding DNA Double-Strand Break Repair Systems in Mycobacteria
Source: PLoS One. 2012 Dec 10;7(12):e51064. doi: 10.1371/journal.pone.0051064 (PMC3519483; doi:10.1371/journal.pone.0051064)
Supplement: Table S2 — List of plasmids, general description, genetic markers and sources/references. (RTF) [file pone.0051064.s009.rtf]

Table S2. List of plasmids, general description, genetic markers and sources/references.
Plasmid	General description	Genetic markers	Source or reference	
Cloning vectors	
pMV206Km	E. coli-Mycobacterium shuttle vector	KmR	Med-Immune Inc., Gaithersburg, MD.	
pMV306Km	Mycobacterial integration vector carrying a mycobacetriophage L5 site-specific recombination system encoding the integrase and containing attP site; it self-replicates in E. coli	KmR	Med-Immune Inc., Gaithersburg, MD.	
pUCH	Source of hph (HygR) gene	AmpR HygR	UTHCT1 Texas, USA	
pINT3	Source of aacC1 (GmR) gene	GmR	UTHCT1 Texas, USA	
pGOAL17	Source of lac Z (blue color) gene under mycobacterial Pag 85 promoter control	AmpR, lac Z	[1]	
p2NIL	Mycobacterial recombination delivery vector, nonreplicating in mycobacteria	AmpR, KmR	[1]	
pJET1.2	Cloning vector	AmpR	Fermentas	
Vectors used for evaluating the stability of DRs and IRs in E. coli and M. smegmatis	
pCE	pMV206Km with hph (HygR) gene and aacC1 (GmR) gene cloned at EcoRI site inside hph (HygR) 	KmR, HygS, GmR	This study	
pCI	pMV306Km with hph (HygR) gene and aacC1 (GmR) gene cloned at EcoRI site inside hph (HygR) gene	KmR, HygS, GmR	This study	
pIREA	pMV206Km with hph (HygR) gene which was disrupted by two IRs introduced within EcoRI site. IRs were separated by aacC1 (GmR) gene cloned in orientation A. The disrupted hph was also flanked, 5' and 3' respectively, by the aph (KmR) and lacZ (blue color) genes. 	KmR, HygS, GmR, lac Z	This study	
pIREB	pMV206Km with hph (HygR) gene which was disrupted by two IRs introduced within EcoRI site. IRs were separated by aacC1 (GmR) gene cloned in orientation B. The disrupted hph was also flanked, 5' and 3' respectively, by the aph (KmR) and lacZ (blue color) genes.	KmR, HygS, GmR, lac Z	This study	
pDREA	pMV206Km with hph (HygR) gene which was disrupted by DRs introduced within EcoRI site. DRs were separated by aacC1 (GmR) gene cloned in orientation A. The disrupted hph was also flanked, 5' and 3' respectively, by the aph (KmR) and lacZ (blue color) genes.	KmR, HygS, GmR, lac Z	This study	
pDREB	pMV206Km with hph (HygR) gene which was disrupted by DRs introduced within EcoRI site. DRs were separated by aacC1 (GmR) gene cloned in orientation B. The disrupted hph was also flanked 5' by the aph (KmR) gene.	KmR, HygS, GmR	This study	
pIRIA	pMV306Km with hph (HygR) gene which was disrupted by two IRs introduced within EcoRI site. IRs were separated by aacC1 (GmR) gene cloned in orientation A. The disrupted hph was also flanked, 5' and 3' respectively, by the aph (KmR) and lacZ (blue color) genes.	KmR, HygS, GmR, lac Z	This study	
pIRIB	pMV306Km with hph (HygR) gene which was disrupted by two IRs introduced within EcoRI site. IRs were separated by aacC1 (GmR) gene cloned in orientation B. The disrupted hph was also flanked, 5' and 3' respectively, by the aph (KmR) and lacZ (blue color) genes.	KmR, HygS, GmR, lac Z	This study	
pDRIA	pMV306Km with hph (HygR) gene which was disrupted by DRs introduced within EcoRI site. DRs were separated by aacC1 (GmR) gene cloned in orientation A. The disrupted hph was also flanked, 5' and 3' respectively, by the aph (KmR) and lacZ (blue color) genes.	KmR, HygS, GmR, lac Z	This study	
pDRIB	pMV306Km with hph (HygR) gene which was disrupted by DRs introduced within EcoRI site. DRs were separated by aacC1 (GmR) gene cloned in orientation B. The disrupted hph was also flanked, 5' and 3' respectively, by the aph (KmR) and lacZ (blue color) genes.	KmR, HygS, GmR, lac Z	This study	
Vectors used for engineering the ÄrecBCD M. smegmatis strain 	
pMK208	pJET carried the region upstream from recC gene together with its 5' fragment	AmpR	This study	
pMK209	pJET carried the region downstream of recD gene with its 3' fragment	AmpR	This study	
pMK210	p2NIL carried the region downstream of recD gene with its 3' fragment	KmR	This study	
pMK211	p2NIL carried the region upstream from recC gene together with its 5' and the region downstream of recD gene with its 3' fragment 	KmR	This study	
pMK212	pMK211 with PacI cassette from pGOAL17; pMK212 is a suicide delivery vector	KmR	This study	

1 UTHCT, The University of Texas Health Center at Tyler

References
1. Parish T, Stoker NG (2000) Use of a flexible cassette method to generate a double unmarked Mycobacterium tuberculosis tlyA plcABC mutant by gene replacement. Microbiology 146 ( Pt 8): 1969-1975.
