# Supplementary material for: Direct and Inverted Repeats Elicit Genetic Instability by Both Exploiting and Eluding DNA Double-Strand Break Repair Systems in Mycobacteria
Source: PLoS One. 2012 Dec 10;7(12):e51064. doi: 10.1371/journal.pone.0051064 (PMC3519483; doi:10.1371/journal.pone.0051064)
Supplement: Table S3 — Primer sequences used for PCR amplification. (RTF) [file pone.0051064.s010.rtf]

Table S3. Primer sequences used for PCR amplification.
Primer	Sequence	Amplified region	
hygF	5'-AACTGCGCCAGTTCCTCCGG-3'	hph (HygR) gene	
hygR	5'-ATTCCTACGCGAGCCTGCGG-3'		
gmF	5'-ATGTTACGCAGCAGCAACG-3'	aacC1 (GmR) gene	
gmR	5'-TTAGGTGGCGGTACTTGGG-3'		
MSattBF	5'-GCTGGCCGTATTGAACTTG-3'	attB site of chromosomal DNA of mycobacteria	
MSattBR	5'-TGATGTCTTTCTCGGTGTCC-3'		
HYGF	5'-GTATTGATTCGGATGATTCC-3'	75-nucleotide sequences comprising two IRs or DRs within EcoRI site of the hph (HygR) gene	
hygr	5'-ccgagctcctccagggctcg-3'		
MSrecBCDGR1 KpnI	5'-ggggtaccccgaacctgatccgcgaggg-3'	Upstream region of M. smegmatis recC gene together with its 5' end	
MSrecBCDGR2 HindIII	5'-gcaagctttgtcctcacgtcggggcagc-3'		
MSrecBCDGR3 HindIII	5'-gcaagcttctcggtgaccagcgtgaggc-3'	Downstream region of M. smegmatis recD gene together with its 3' end	
MSrecBCDGR4 PstI	5'-aactgcagcgcgctcaactgagttcccc-3'		
MSrecBCDGR3 HindIII	5'-gcaagcttctcggtgaccagcgtgaggc-3'	Downstream region of M. smegmatis recD gene together with its 3' end used as a probe in Southern blot	
MSrecBCD rev probe	5'-ggacgggcggtctaaacccc-3'		
