# Supplementary material for: Direct and Inverted Repeats Elicit Genetic Instability by Both Exploiting and Eluding DNA Double-Strand Break Repair Systems in Mycobacteria
Source: PLoS One. 2012 Dec 10;7(12):e51064. doi: 10.1371/journal.pone.0051064 (PMC3519483; doi:10.1371/journal.pone.0051064)
Supplement: Table S4 — Criteria used for in silico analyses of repetitive elements in bacterial genomes. (RTF) [file pone.0051064.s011.rtf]

Table S4. Criteria used for in silico analyses of repetitive elements in bacterial genomes.
Repeat elements	Criteria for genome scanning	Potential non-B DNA conformations	
Tandem repeats, 1-4 nucleotide (nt) units	≥12 nt	Multiple1	
Tandem repeats, 5-14 nt units	≥2 repeats	Multiple1	
Direct repeats	Any sequence ≥15 nt repeated anywhere in the genome but not in tandem	Looped structures	
Inverted repeats	Two tracts ≥10 nt with same sequence on the complementary strands, separated by up to half tract length but no more than 20 nt	Cruciforms	
(RY)n tracts	Two RY tracts with mirror symmetry, >12 nt each and separated by 1-10 nt 	Triplexes	
Tetraplex-forming motifs	Four runs of at least 3 Gs (or 3 Cs), each separated by 1-7 nt	Tetraplexes	

1 Slipped/hairpin structures, cruciforms, triplexes, and tetraplexes, depending on sequence composition.
R, purines (A, G); Y, pyrimidines (C, T). Search criteria did not allow for any mismatches within the repetitive motifs.
