# Supplementary material for: Direct and Inverted Repeats Elicit Genetic Instability by Both Exploiting and Eluding DNA Double-Strand Break Repair Systems in Mycobacteria
Source: PLoS One. 2012 Dec 10;7(12):e51064. doi: 10.1371/journal.pone.0051064 (PMC3519483; doi:10.1371/journal.pone.0051064)
Supplement: Table S5 — Statistics of in silico searches of repeats genome-wide. (RTF) [file pone.0051064.s012.rtf]

Table S5. Statistics of in silico searches of repeats genome-wide.
Genome	Genome length [bp]	Variable1	Direct repeats2	Inverted repeats3	Tandem repeats of	RY mirror repeats	Tetraplex-forming motifs	
					1 to 4 bases	> 4 bases			
E. coli K12	4,639,675	Total
Percent
Ratio
Mean
Max	67189
29.1
4.5
20.1
2815	705
0.46
9.8
30.1
56	119
0.03
1.8
12.3
18	5926
1.5
1.3
11.7
425	51
0.01
1.1
12.6
16	52
0.03
0.6
23.7
32	
M. smegmatis mc2155	6,988,209	Total
Percent
Ratio
Mean
Max	600017
140.8
3.4
16.4
55473	944
0.43
2.9
31.8
67	383
0.07
1.2
12.4
17	14371
2.5
1.3
12.0
360	344
0.06
1.0
12.7
18	331
0.12
0.2
24.7
33	
M. tuberculosis H37Rv	4,411,532	Total
Percent
Ratio
Mean
Max	201236
77.4
3.8
17.0
1697	498
0.38
3.0
33.4
70	352
0.10
2.1
12.8
22	8416
2.6
1.3
13.7
1473	98
0.03
0.5
12.7
17	426
0.25
0.4
25.6
33	

1 Total, total number of repeats observed genome-wide; Percent, percentage of genome occupied by the repeats. The percent data represent the total number of bases in the repeats divided by the total number of bases in the genome. However, because, every identified sequence was considered separately, the percent may be more than 100 for overlapping motifs. Ratio, ratio of the Total to the predicted number of repeats found in the randomized genomes after reshuffling. Mean, mean length of the repeats. Max, maximum length of repeats.
2 The repeat units without the intervening sequence separating them were considered.
3 The repeat units with the intervening sequence separating them were considered.
